# Association of radiomic features with genomic signatures in thyroid cancer: a systematic review

**DOI:** 10.1186/s12967-024-05896-z

**Published:** 2024-11-30

**Authors:** Neila Luciano, Francesca Maria Orlandella, Mariantonia Braile, Carlo Cavaliere, Marco Aiello, Monica Franzese, Giuliana Salvatore

**Affiliations:** 1 Dipartimento di Scienze Biomediche Avanzate, Università degli Studi di Napoli “Federico II”, Naples, Italy; 2 Dipartimento delle Scienze Mediche, Motorie e del Benessere, Università Degli Studi di Napoli Parthenope, Naples, Italy; 3 CEINGE Biotecnologie Avanzate Franco Salvatore S.c. a r.l., Naples, Italy; 4 IRCCS SYNLAB SDN, Naples, Italy

**Keywords:** Thyroid cancer, Radiomics, Genomics, Metastasis, RET/PTC, BRAF^V600E^

## Abstract

**Background:**

There is a growing interest on the association of radiomic features with genomic signatures in oncology. Using computational methods, quantitative radiomic data are extracted from various imaging techniques and integrated with genomic information to construct predictive models aimed at advancing diagnostic strategies in cancer patient management. In this context, the aim of this systematic review was to assess the current knowledge on potential application of this association in patients with thyroid cancer (TC).

**Methods:**

A comprehensive literature review was conducted by querying three different databases (PubMed, Scopus and Embase) to identify studies published until June 2024, focusing on the potential association of radiomics and genomics in patients with TC. Pertinent data were subsequently extracted, and the methodological quality was evaluated using the A MeaSurement Tool to Assess Systematic Reviews 2 (AMSTAR 2).

**Results:**

From the initial analysis, a total of 853 papers were identified. After removing duplicates and applying eligibility criteria, we ultimately evaluated 7 articles. It was observed that the most commonly utilized imaging technique for TC examination was ultrasound (US), followed by computed tomography and magnetic resonance imaging. Regarding genomic techniques, sequencing and polymerase chain reaction were the most commonly employed methods to validate genetic alterations. The association of radiomic features with genomic signatures demonstrated promising performance in predicting metastasis to the cervical lymph nodes or RET/PTC rearrangements. The effectiveness of models based on US-radiomic features in predicting BRAF^V600E^ mutation in patients with TC requires further investigation.

**Conclusion:**

Although this systematic review has several limitations, primarily related to the limited amount of available literature data, the association of radiomic features with genomic signatures demonstrates a potential as non-invasive tool to enhance the accuracy and efficacy of TC diagnosis and prognosis.

*PROSPERO registration number*: CRD42024572292.

**Supplementary Information:**

The online version contains supplementary material available at 10.1186/s12967-024-05896-z.

## Introduction

Among the malignancy of the endocrine system, thyroid cancer (TC) represents the primary disease with a survival rate of approximately 99% [1]. Most TC arise from follicular cells, while a small percentage (less than 5%) is represented by medullary thyroid carcinomas which originate from parafollicular or C cells [2, 3].

Papillary thyroid carcinoma (PTC), the most prevalent form of TC, typically exhibits a favorable prognosis [4, 5]. Nevertheless, there are still cases of high-risk PTC patients who may experience recurrence and metastasis, mainly located at the central and lateral neck lymph nodes [6, 7].

The diagnosis of PTC and its associated lymph node metastases involves fine-needle aspiration, a widely utilized technique for sampling thyroid nodules and assessing suspicious lymph nodes [6, 8].

Genomic sequencing techniques unveiled a wide range of targetable genetic alterations in the pathogenesis of TC [9–11]. One of these genetic aberrations implicated in the development of PTC is the RET chromosomal rearrangement [12]. Additionally, mutations of the RAS gene are molecular events that play a role in the pathogenesis of PTC, leading to constitutive activation of downstream signaling pathways, such as the mitogen-activated protein kinase pathway [13, 14]. Another key genetic alteration in PTC involves mutations in the B-Raf proto-oncogene (BRAF), notably the V600E mutation [15], crucial for the molecular classification of PTC [16, 17] and targeted therapeutic approaches [18, 19]. Indeed, BRAF^V600E^ mutation is associated with tumor aggressiveness and increased propensity for metastasis [4, 20–22].

The main therapeutic treatments for TC include thyroid surgery followed by radioactive iodine-131 (^131^I) ablation and hormone suppression therapy [23]. Subsequently, the patient's follow-up consists of periodic measurements of serum thyroglobulin (Tg), serum thyroid-stimulating hormone (TSH), and Tg antibodies, along with monitoring through imaging methods such as neck ultrasonography (US) [18]. US is considered the most sensitive tool for detecting locally recurrent disease [23, 24].

Other recommended imaging methods include computed tomography (CT) and magnetic resonance imaging (MRI) for evaluating tumor burden with neighbor structures [25, 26], and whole-body hybrid scan (WBS) such as in combination with single photon emission tomography/CT (SPECT/CT) [27], and/or positron emission tomography/CT (PET/CT), recommended in the follow-up to localize any metastatic disease [6, 28].

The field of medical image analysis has expanded by introducing processes aimed at extracting quantitative features from images using data characterization algorithms [29]. This workflow, known as radiomics, starts with the definition of region of interests (ROIs) on high-resolution images, and returns as output quantitative features, categorized into shape-based, first-, second-, and high-order metrics [30].

The integration of radiomic features with genomic signatures is a dynamic and promising field that has gained considerable attention in cancer research and in clinical practice. Indeed, the combination of radiomics and genomics, namely radiogenomics, has the goal of unravel the relationship between quantitative information obtained from radiological imaging and genomic alterations within cancer cells across different scales [31, 32], improving diagnosis [33], patients’ outcomes [34], and uncovering patterns relevant to a patient's prognosis [35].

In consideration of these assumptions, this systematic review was conducted with the purpose of evaluating the current knowledge on the possible application of associating radiomic features with genomic signatures in individuals affected with TC.

## Methods

### Search strategy

This study was registered on the PROSPERO database number CRD42024572292 (available from https://www.crd.york.ac.uk/prospero/display_record.php?ID=CRD42024572292).

The literature search was conducted on three different databases, PubMed, Scopus and Embase, using the following combination of keywords:

(((((radiomics[Title/Abstract]) OR (radiogenomics[Title/Abstract])) OR (genomics[Title/Abstract])) OR (transcriptomics[Title/Abstract])) OR (omics[Title/Abstract])) AND (thyroid cancer[Title/Abstract]);

(((((radiomics[Title/Abstract]) OR (radiogenomics[Title/Abstract])) OR (genomics[Title/Abstract])) OR (transcriptomics[Title/Abstract])) OR (omics[Title/Abstract])) AND (thyroid carcinoma[Title/Abstract]).

The search is updated on June 25, 2024.

To assess the eligibility of each article, each PICO (Population, Intervention, Comparison, Outcome) element was identified as follows: Population (P): subjects (human) with thyroid cancer; Intervention (I): imaging modalities (e.g., CT, MRI, PET/CT, US) and genomic techniques (e.g., sequencing, PCR); Comparison (C): imaging features with genomic information (presence/absence of specific mutation) or with presence/absence of metastasis in TC patients; Outcome (O): diagnostic effectiveness of imaging methods to predict genomic features or the insurgence of recurrence or metastasis in TC patients.

The various topics analyzed in this systematic review are summarized in a checklist (Additional file 1) according to the Preferred Reporting Items for Systematic Reviews and Meta-Analyses (PRISMA) 2020 statement [36].

### Inclusion and exclusion criteria

One author searched the combination of keywords using the Boolean operators AND and OR across three different databases. For each search result, the author merged the findings into an electronic spreadsheet. After eliminating duplicates, the author combined the three spreadsheets into a single electronic spreadsheet, further removing duplicates. Afterwards, a second author removed any remaining duplicates. Then, independently, three authors screened each remaining article based on the following inclusion criteria: (a) studies focused on patients affected by thyroid cancer; (b) studies based on imaging techniques as predictors of genomic signatures; (c) studies related to gene expression analysis as predictors of imaging biomarkers; and (d) studies on human.

Regarding the exclusion criteria (Additional file 2), articles were considered ineligible if they fell into any of the following categories: (a) review, systematic review, or meta-analysis; (b) book chapter or editorial; (c) conference abstract or conference paper; (d) no English language; (e) full-text not available; (f) the study only included imaging or genomic expression; and (g) non-human subjects (e.g., animals).

In cases of disagreement among the three authors, a fourth author intervened to assess the articles.

### Data extraction and quality assessment

Following the screening process, 7 articles emerged for the final analysis. Thus, three authors extracted and summarized the data from each article.

A quality assessment for the case–control studies utilizing the Newcastle–Ottawa Scale (NOS) assessment tool, was conducted [37]. The case–control study form includes eight elements divided into three domains: selection, comparability and exposure (with a score ranging from 0 to 9 points). A higher score correlates with a superior quality of the study. Resulting, the risk of bias for each study was assessed based on three questions related to eligible articles: (1) Bias related to the clinical information of enrolled patients; (2) Bias related to the radiomics features extracted from imaging techniques; (3) Bias related to the ascertainment of exposure. Three authors independently reviewed each article. Each item was categorized as “low risk of bias” when the score exceeded 7; otherwise, it was considered at a “high risk of bias” [38].

To assess the methodological quality of this systematic review, we addressed 16 criteria outlined in A MeaSurement Tool to Assess systematic Reviews 2 (AMSTAR 2) [39] reported in Additional file 3. The 16 items examine different critical and non-critical domains. Specifically, critical domains are related to protocol registration, suitability literature search, justification for exclusion of studies, risk of bias from individual studies, adequacy of meta-analytic methods and heterogeneity assessment.

AMSTAR 2 tool does not assign a numeric score to the systematic review, however, based on the presence of critical and non-critical flaws, it categorizes systematic reviews into four levels of confidence (high, moderate, low and critically low).

## Results

### Literature research

**Fig. 1:**
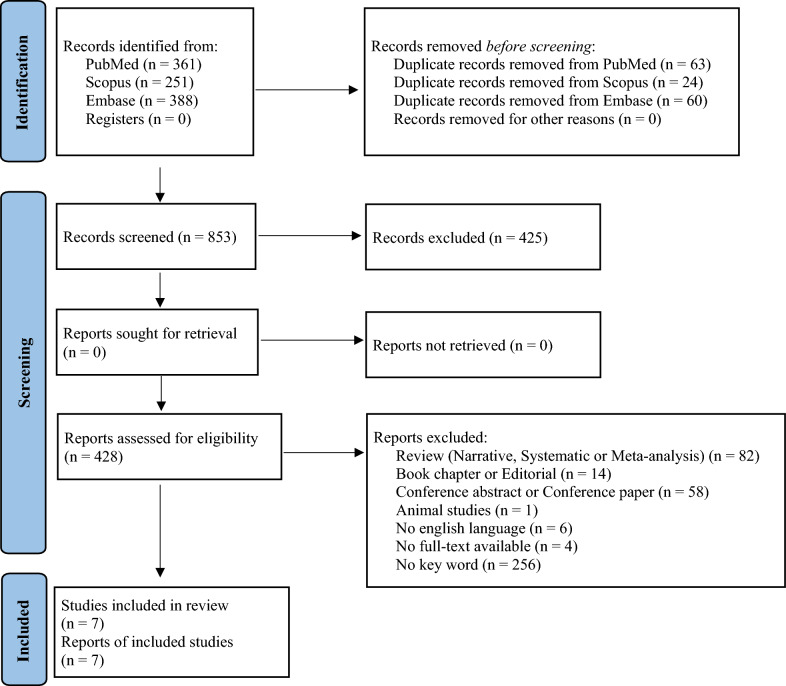
PRISMA flow-diagram showing research strategy [36]

A total of 853 studies were identified from the initial search on PubMed, Scopus, and Embase. After removing duplicates, 428 articles were assessed against the above-mentioned inclusion and exclusion criteria. Finally, 7 studies were found to be eligible for the final analysis (Fig. 1).

### Study characteristics

The main characteristics of all the included studies are reported in Table 1. All the included studies were published between 2020 and 2024 and were conducted on human subjects. Furthermore, 1 study was prospective [40], 5 were retrospective [41–45] and 1 was a prospective and retrospective multicenter study [46]. The number of subjects enrolled in the studies varied from 80 to 650, with a majority of females. All the studies selected were conducted on PTC patients.

**Table 1:** Characteristics and outcome of the studies on PTC patients assessed for eligibility criteria

Author, year	Study design	Enrolled population, *n*	Imaging modalities, *n*	Genomic techniques, *n*	Outcome
Dong et al. 2023 [46]	Prospective and retrospective multicenter study	471 patients: Males, N/A Females, N/A	CT (*n* = 471)	Transcriptome sequencing (*n* = 14)	Good predictive value of CT-radiomics features to predict LNLN
Tong et al. 2021 [40]	Prospective study	270 patients: Males, 83 Females, 187	US (*n* = 270)	LC–MS/MS (*n* = 49); IHC (*n* = 40)	Good performance of US-radiomic signature to predict the CLN status
Wang et al. 2022 [42]	Retrospective study	138 patients: Males, 51 Females, 87	US (*n* = 138)	PCR (*n* = 138)	Good predictive value of gray-scale US to predict BRAF^V600E^ mutation
Yoon et al. 2020 [43]	Retrospective study	527 patients: Males, 122 Females, 405	US (*n* = 527)	DNA sequencing (*n* = 527)	Limited value of US-radiomic features to predict BRAF^V600E^ mutation
Yu et al. 2022 [44]	Retrospective study	650 patients: Males, 145 Females, 505	US (*n* = 650)	NGS; ARMS RT-PCR; Sanger sequencing (*n* = 650)	Good predictive value of US-based DLRN to predict RET rearrangement
Zhang et al. 2024 [45]	Retrospective study	182 patients	US (*n* = 108)	NGS (*n* = 182)	Good predictive value of genetic alteration and US-radiomic features to predict the prognosis
Zheng et al. 2023 [41]	Retrospective observational study	80 patients: Males, 17 Females, 63	MRI (*n* = 80)	ARMS RT-PCR; Sanger sequencing (*n* = 80)	Good predictive value of MRI-radiomics features to predict BRAF^V600E^ mutation

*ARMS RT-PCR* Amplification Refractory Mutation System Real Time-Polymerase Chain Reaction, *CLN* Cervical Lymph Node, *CT* Computed Tomography, *DLRN* Deep Learning Radiomics Nomogram, *IHC* Immunohistochemistry, *LC–MS/MS* Liquid Chromatograph-Mass Spectrometry, *LNLN* Lateral Neck Lymph Node, *MRI* Magnetic Resonance Imaging, *n* Number, *N/A* Not available, *NGS* Next Generation Sequencing, *PTC* Papillary Thyroid Cancer, *US* Ultrasonography

Additional file 4 summarizes the radiomic features extracted, imaging modalities used for each study.

Based on the outcomes of each study, we grouped the analyzed articles as follows: studies in which the association of radiomic and genomic data was fulfilled to predict metastasis (3 studies); studies in which the association was exploited to predict genetic alterations in TC (4 studies).

### Clinical outcome

#### Predicting metastasis

PTC can metastasize to the cervical lymph nodes (CLN) and its diagnosis plays a crucial role in planning surgical method, therapeutic treatment and in evaluating the prognosis of PTC patients [47–49].

In this review, we identified 3 studies suggesting that an association-based approach could significantly improve the predictive detection of CLN involvement in PTC.

The study of Dong et al*.* [46] involved a multicenter approach, including 1298 lateral neck lymph nodes (LNLNs) from PTC patients who underwent preoperative computed tomography (CT) examinations, open surgery, and lateral neck dissection. The LNLNs were divided into training cohort (*n* = 800), internal test cohort (*n* = 345), prospective test cohort (*n* = 85) and external test cohort (*n* = 68) (Table 2). Radiomics features were extracted from both contrast and non-contrast CT images, and a radiomics signature (Rad-score) (Additional file 4) was combined with clinical risk factors in a nomogram, demonstrating robust prognostic ability in predicting LNLN metastasis. Subsequently, transcriptomics sequencing was carried out to investigate the biological basis of the nomogram's predictions. For this purpose, 14 tumor samples were collected for next-generation RNA-seq and grouped into high and low-risk categories by the nomogram. Gene expression analysis revealed significant differences between these risk groups, particularly emphasizing the ribosome as the most significant term in biological processes, suggesting ribosome-targeted therapy as a promising approach at this stage.

**Table 2:** Study designs and the main clinicopathological characteristics of PTC patients included for predicting metastasis

Author, year	Comparison group, *n*		Gender, *n*	Age < 55/ ≥ 55,* n* or range of years	Tumor size (cm) range or ≤ 1/ > 1, *n*	Tumor staging, *n*	Metastasis location	Type of surgery
Dong et al. 2023 [46]	Training set, 800	Metastasis, 497 Non-Metastasis, 303	Male, 568 Female, 232	< 55, 184 ≥ 55, 616	0.4–6.7	N/S	LNLN	Open surgery and lateral neck dissection
	Test set: Internal, 345 External, 68 Prospective, 85	Metastasis, 331 Non-Metastasis, 167	Male, 145 Female, 353	< 55, 215 ≥ 55, 283	0.3–6.3	N/S	LNLN	
Tong et al. 2021 [40]	Training set, 180	Metastasis, 81	Male, 30 Female, 51	20–68	0.4–3.0	N/S	CLN	Lobectomy/thyroidectomy and central CLNs dissection
		Non-Metastasis, 99	Male, 24 Female, 75	18–65	0.3–2.3			
	Validation set, 90	Metastasis, 43	Male, 17 Female, 26	23–65	0.5–2.6	N/S	CLN	
		Non-Metastasis, 47	Male, 12 Female, 35	25–63	0.3–2.0			
Zhang et al. 2024 [45]	Training Set, 76	PTC cancer tissues, 124	Male, 30 Female, 94	< 55, 94 ≥ 55, 30	≤ 1, 63 > 1, 61	Stage I, 118 Stage II, 2 Stage III,4	LN	FNA
	Test Set, 32	Benign thyroid nodules, 58	Male, 14 Female, 44	< 55, 43 ≥ 55, 15	N/A	N/A	N/A	

*CLN* Cervical Lymph Node, *FNA* Fine-Needle Aspiration, *LN* Lymph Node, *LNLN* Lateral Neck Lymph Node, *n* Number, *N/A* Not Applicable, *N/S* Not Specified

Likewise, Tong et al*.* [40], investigated a novel radiomics approach to predict CLN status in PTC by correlating radiomics information obtained from preoperative thyroid ultrasound (US) with the molecular characteristics of the patients. To this end, 270 patients were enrolled, divided into training cohort (*n* = 180 patients) and validation cohort (*n* = 90 patients) (Table 2). Patients underwent either lobectomy or total thyroidectomy, with routine central CLNs dissection. Radiomics features demonstrated superior predictive accuracy (82.2% and 80.0% in the training and validation cohorts, respectively) compared to conventional US examination (60.6% and 63.3%). The authors then identified gene modules associated with CLN metastasis (CLNM) through iTRAQ assessment and LC–MS/MS, followed by ROC analysis. Specifically, 49 tumor samples were collected during lymph node removal, and proteins were subsequently isolated. Weighted gene co-expression network analysis (WGCNA) generated sixteen gene modules, with eight showing good discriminatory performance (AUC > 0.5) for CLNM prediction. Finally, 28 radiomic features selected showed significant correlations with gene modules in a radiogenomic map, mainly sustained by GO and KEGG pathways. Moreover, immunohistochemical staining confirmed the involvement of the hub genes LAMC1 and THBS, both included in the PI3K/AKT signaling pathway, demonstrating significant differences between PTC tissues with and without CLNM.

The last selected study for this section speculates about the association of next-generation sequencing (NGS) with US-based radiomics as a tool to enhance the predictive accuracy of benign and malignant thyroid nodules [45]. Using NGS, a total of 592 mutations were identified among 31 genes by analyzing both PTC tissues and benign nodules. The mutations were spread across 13 driver genes (e.g., BRAF, RET, TP53) and 18 non-driver genes (e.g., CHEK2, ATM, BRCA1), identifying mutations in BRAF (i.e., BRAF^V600E^), TERT and RET as more frequently associated in cancer tissues than benign nodules, and highly associated with lymph node metastasis. In addition, to enhance predictive accuracy, the authors combined genetic data with clinical features and radiomic signatures derived from US images (Additional file 4). To this aim, the patients (*n* = 108) were randomly assigned to training and test sets (Table 2), and an integrative nomogram model was constructed. This integrative model showed higher efficacy in predicting lymph node metastasis compared to models based on individual data types, highlighting the importance of integrating various data types for a more accurate prediction of lymph node metastasis in PTC.

#### Predicting genetic alteration

Developing diagnostic methods capable of predicting the presence of genetic mutations associated with PTC may guide therapeutic strategies, with a less invasive approach based on the so called “virtual biopsy”.

In this review, we examined 4 articles analyzing the ability of radiomics-based models to predict BRAF^V600E^ mutation or RET oncogenic rearrangement in PTC. Among the 4 articles, 3 of them focused on assessing the utility of radiomic features in predicting BRAF^V600E^ mutation and 1 focus on RET rearrangement.

An interesting study was performed by Zheng et al. [41] that conducted an evaluation to assess the feasibility of preoperatively identifying BRAF^V600E^ mutations in PTC using an MRI-based texture feature model. To this aim, 80 patients with PTC were enrolled and divided into BRAF^V600E^ mutant (*n* = 58) and BRAF^V600E^ wild-type (*n* = 22) groups (Table 3) after Sanger sequencing. Initially, 1132 texture features were extracted from two MRI parameters: T2-weighted imaging (T2WI) and contrast-enhanced T1-weighted imaging (CE-T1WI) (Additional file 4). Subsequent analyses, involving univariate logistic regression and minimum redundancy maximum relevance (mRMR) algorithm, lead to the identification of 6 features for the T2WI model, 7 for the CE-T1WI model, and 7 for the combined model (a fusion of the T2WI and CE-T1WI models), all relevant to the BRAF^V600E^ mutation. These features were then subjected to multivariate logistic regression, resulting in 4 predictors for T2WI, 4 for CE-T1WI, and 5 for the combined model. All three MRI models (T2WI, CE-T1WI, and combined) demonstrate a strong predictive ability for mutation status, with a higher accuracy, sensitivity, and negative predictive value for the combined one.

**Table 3:** Study designs and the main clinicopathological characteristics of PTC patients included for predicting genetic alteration

Author, year	Comparison group, *n*		Gender, *n*	Age (mean ± SD, years)	Tumor size (mean ± SD, mm)	Tumor Staging	Tumor location, *n*	Genomic detection method
Zheng et al. 2023 [41]	Training set, N/S Test set, N/S	BRAF^V600E^, 58	Male, 11 Female, 47	43.31 ± 11.95	12.16 ± 7.16	N/S	Left lobe, 26 Right lobe, 32	Sanger sequencing
		BRAF_WT, 22	Male, 6 Female, 16	43.73 ± 15.22	11.78 ± 6.86	N/S	Left lobe, 10 Right lobe, 12	
Wang et al. 2022 [42]	Training set, 96 Validation set, 42	BRAF^V600E^, 63	Male, 22 Female, 41	38.03 ± 10.41	24.12 ± 8.6	N/S	N/S	PCR
		BRAF_WT, 75	Male, 29 Female, 46	36.68 ± 10.05	23.98 ± .11.01	N/S	N/S	
Yoon et al. 2020 [43]	Training set, 387 Validation set, 140	BRAF^V600E^, 428	Male, 326 Female, 102	42.7 ± 13.8	16.0 ± 7.6	N/S	N/S	DNA sequencing
		BRAF_WT, 99	Male, 79 Female, 20	38.4 ± 13.2	18.0 ± 9.1	N/S	N/S	
Yu et al. 2022 [44]	Training set, N/S Test set: N/S	RET/PTC rearrangement, 103	Male, 15 Female, 88	37.9 ± 11.2	13.6 ± 7.9	N/S	Upper pole, 22 Lower pole, 33 Middle, 48	NGS
		Non-RET/PTC rearrangement, 547	Male, 130 Female, 417	43.7 ± 11.0	8.5 ± 6.4	N/S	Upper pole, 140 Lower pole, 188 Middle, 219	

*n* Number, *NGS* Next Generation Sequencing, *N/S* Not Specified, *PTC* Papillary Thyroid Cancer, *WT* Wild Type

Similarly, in the retrospective study by Wang et al*.* [42], 138 PTC patients that underwent preoperative thyroid US examination, surgery and BRAF^V600E^ mutation detection through PCR were examined. Patients were divided into the BRAF^V600E^ mutation-free group (*n* = 75) and the BRAF^V600E^ mutation group (*n* = 63) (Table 3) and then randomly assigned to the training group (*n* = 96) or the test group (*n* = 42). A comprehensive set of 479 radiomic features was obtained from US images and through the application of advanced algorithms (Additional file 4), these features related to BRAF^V600E^ mutation were reduced to 8 for grayscale and 5 for elasticity US. Three different radiomic models were constructed based on grayscale features, elastic features and the combination of both features, revealing that the combination of grayscale and elasticity performed better than using grayscale alone, and suggesting a potential added value for elasticity US radiomics.

Moreover, the performance of US-based radiomic models in predicting the BRAF^V600E^ mutation in PTC patients was evaluated by Yoon et al*.* [43]. A total of 527 patients, who underwent thyroid surgery with BRAF mutation status detection through DNA sequencing, were divided into either the training cohort (*n* = 387) or the validation cohort (*n* = 140). Of these, 428 were positive and 99 were negative for BRAF^V600E^ mutation (Table 3). Notably, patients with BRAF^V600E^ mutation had significantly smaller tumors. Given that the frequency of the BRAF^V600E^ mutation correlates with tumor size and conventional PTC, a subgroup of patients (*n* = 389) with confirmed conventional PTC measuring < 20 mm was analyzed. Radiomics analysis was conducted and 730 features were collected (Additional file 4). From these, 8 potential features were selected in the training cohort, and utilized to calculate the Radiomics Score. Regarding conventional PTC, 4 radiomic features were chosen from the 730 total features, forming the basis for the Radiomic Score calculation. In both total TC and the conventional PTC subgroup, the Radiomics Score shows a significant association with the presence of BRAF^V600E^ mutation on multivariable analysis. Although the results showed a good discriminatory ability for both the total TC and the conventional PTCs < 20 mm training sets, this trend was not consistently confirmed in the respective validation sets.

As previously mentioned, radiomic features can also be utilized to predict oncogenic rearrangements, providing physicians a non-invasive primary screening method, thereby improving treatment outcomes. The last selected paper [44] aimed to evaluate the ability of a deep learning radiomics nomogram (DLRN) based on US images to preoperatively predict RET rearrangement in PTC. To achieve this, 650 patients with PTC were enrolled, divided into the RET/PTC rearrangement group (*n* = 103) and the non-RET/PTC rearrangement group (*n* = 547) (Table 3), and then assigned to the training and test cohorts using a fivefold cross-validation method. Chromosomal aberration presence of RET was assessed through next-generation sequencing. Feature extraction included 1477 hand-crafted features from US images and 128 deep transfer learning (DTL) features (Additional file 4). A logistic regression model was used to create the DLRN combining clinical and radiomic signatures. The nomogram exhibited superior predictive performance in both training and test cohorts compared to other models, suggesting that a DLRN, combining deep learning and traditional US features can increase the diagnostic power for RET rearrangement in PTC.

### Quality assessment and risk of Bias across studies

The risk of bias was systematically appraised across individual studies using the Newcastle–Ottawa Scale (NOS) [37], revealing varying degrees of methodological quality (Table 4).

**Table 4:** Newcastle–Ottawa Scale (NOS) for case–control study quality assessment

Authors (year)	NOS selection				NOS comparability	NOS exposure			NOS score
	Case selection	Representativeness of the cases	Control selection	Definition of controls	Comparability of cases and controls	Ascertainment of exposure	Same method of ascertainment for cases and controls	Non-response rate	
Dong et al. (2023)	a	a	a	a**★**	a**★**b**★**	a**★**	a	d	4
Tong et al. (2021)	a**★**	a**★**	a**★**	a**★**	a**★**b**★**	a**★**	a**★**	d	8
Wang et al. (2022)	a**★**	a**★**	a**★**	a**★**	a**★**b**★**	a**★**	a**★**	d	8
Yoon et al. (2020)	a**★**	b	a**★**	a**★**	a**★**b	a**★**	a**★**	d	6
Yu et al. (2022)	a	a**★**	a**★**	a**★**	a**★**b**★**	a**★**	a	d	6
Zhang et al. (2024)	b	b	a	a	a**★**b**★**	a**★**	a**★**	d	4
Zheng et al. (2023)	b	a**★**	a**★**	a**★**	a**★**b**★**	a	a	d	5

**NOS selection**

1) Is the Case Definition Adequate? (a) Yes, the clinical information of the enrolled patients was provided by the hospital (in addition to written informed consent, if available) **★;** (b) yes, the clinical information of the enrolled patients was obtained through self-reports or diagnostic examinations; (c) no description

2) Representativeness of the Cases: (a) yes, the series of cases of interest were representative **★**; (b) potential for selection biases or not stated

3) Selection of Controls: (a) hospital controls **★**; (b) community controls; (c) no description

4) Definition of Controls: (a) yes, the characteristics of controls were well defined (e.g., gender, age, tumor size, tumor location, metastasis location) **★;** (b) no description of source

**NOS comparability**

1) Comparability of Cases and Controls on the Basis of the Design or Analysis: a) imaging techniques such as CT, US, and MRI serve as tools for extracting radiomic features **★;** b) combining radiomic features with genomic signatures resulted to be useful in predicting lymph node metastasis or mutational status in thyroid carcinoma patients **★**

**NOS exposure**

1) Ascertainment of Exposure: (a) secure ascertainment (e.g., imaging examinations, genomic detection techniques) **★;** (b) written self-report; (c) no description

2) Same method of Ascertainment for Cases and Controls: (a) yes **★;** (b) no

3) Non-Response Rate: (a) same rate for both groups **★;** (b) non respondents described; (c) rate different and no designation; (d) no description

Generally, the studies received high scores on the NOS, indicating good quality concerning case selection, comparability, and exposure ascertainment. Key factors contributed to these higher scores include the utilization of representative cases, well-defined controls, and secure methods for ascertaining exposure. The comparability of cases and controls was also a critical factor in the assessment.

In the included studies, NOS scores ranged from 4 to 8, with a mean of 5.86 points (Table 4). According to the established evaluation criteria, 28.6% of the studies achieved a score of 8, indicating high methodological quality and minimal risk of bias. Similarly, another 28.6% of the studies scored 6 indicating a moderate methodological quality with a low risk of bias. Finally, 42.8% of the studies were classified to have a high risk of bias, with a score between 4 and 5.

Upon specific analysis, Tong et al*.* [40] and Wang et al*.* [42] emerge as particularly robust studies, earning high scores in case selection, comparability, and exposure ascertainment. These studies exhibited comprehensive case definitions, representative cases, well-defined controls, and secure methods for exposure ascertainment, contributing to their overall methodological strength.

Conversely, Zheng et al*.* [41] displayed some weaknesses, such as potential bias in the case selection. However, this study compensates with strengths in comparability and exposure ascertainment. The studies of Dong et al. [46] and Zhang et al*.* [45] exhibited a potential risk of bias related to selection and exposure domains. The comparability of cases and controls was adequately addressed by combining radiomic features with genomic signatures, which is a strength of this study.

The articles of Yoon et al*.* [43] and Yu et al*.* [44] were overall robust, demonstrating good quality. Each studies show specific strengths in the selection and definitions of controls and in comparability domain.

While the studies vary in their risk of bias, collectively they emphasize the importance of rigorous case definition, representative cases and well-defined controls to minimize biases and enhance the reliability of findings across diverse research contexts.

Finally, we adopted the 16 items of AMSTAR 2 to critically evaluate the quality of our systematic review; this comprehensive tool evaluates various aspects of the review process, including study selection, data extraction, risk of bias, and reporting standards. The results revealed an overall confidence rating (OCR) of moderate quality. This suggests that our systematic review does not highlight any critical flaw and provides an accurate summary of the results from the included studies.

## Discussion

Investigating the association between radiomic and genomic data is a complex multi-step process that could represent a new approach for the definition of diagnostic, predictive, or prognostic cancer models [31, 32]. One of the fundamental goals of this interdisciplinary approach is to identify specific radiological patterns that correlate with the genomic characteristics of tumors [50].

Using imaging techniques, such as US, MRI, CT, along with sophisticated computational algorithms, clinicians can extract quantitative data from images [30, 51, 52]. The extracted radiomic features serve as a bridge to connect with genomic data, obtained through high-throughput biological techniques (e.g., NGS, Sanger sequencing).

In the context of TC, PTC, the most prevalent variant, generally exhibits a favorable prognosis [4, 5]. However, there are high-risk cases that may undergo recurrence and metastasis, with lymph nodes, both central and lateral, serving as primary sites. Furthermore, PTCs are marked by intratumor heterogeneity, which has a detrimental impact on the prognosis, particularly in advanced tumor stages [53]. Therefore, understanding crucial genetic events in tumorigenesis and correlating them with imaging data, could improve diagnosis and prognosis and thus contribute to a better management of patients.

Based on these assumptions, this systematic review was aimed to highlight the potential applications of the correlation between genomics and radiomics in TC. Through a comprehensive analysis of literature, a total of 7 studies were considered eligible and were grouped into two main categories based on clinical outcomes: predicting metastasis or predicting mutation status.

Thus, in this systematic review the application of radiomics in the prediction of lymph node metastasis was investigated on the basis of the three reports aforementioned [40, 45, 46]. Dong's paper [46] explores a CT radiomics-based nomogram for predicting LNLN metastasis in PTC. There are many strengths of the nomogram obtained from CT such as: (i) the selection of lymph nodes as segmentation objects of research; (ii) the robustness; (iii) stability and performance comparable to senior radiologists. Nevertheless, as highlighted by the authors, there are several limitations: (i) the sample size is too low, (ii) the manual delineation of the ROIs, (iii) the correlation between the CT images with pathological reports can be subjective and uncertain; (iv) experimental validation is needed to support the observation that radiomic features reflect changes in the tumor microenvironment; (v) sequencing was performed on a few tissue biopsies.

The paper of Tong [40] aims to predict CLNM in PTC patients using preoperative thyroid US radiomics and gene modules by WGCNA, highlighting the potential of radiomics compared to the standard imaging assessment, and the correlation with specific gene pathways. Nevertheless, this report has also limitations, such as single-center imaging data and non-compliance to the Imaging Biomarker Standardization Initiative.

The heterogeneity of PTC, characterized by various gene mutations and fusions, necessitates advanced diagnostic tools for better treatment planning. NGS has become instrumental in identifying these genetic changes, which are crucial for predicting aggressive cancer phenotypes.

In the study by Zhang and colleagues [45], NGS was employed to compare genetic mutations (e.g., BRAF^V600E^) in PTC tissues and benign nodules. The authors found that combining gene alterations with ultrasound-related radiomics features improved predictions of lymph node metastasis. Despite these promising findings, the study has several limitations, including a sample size that may not be large enough to generalize the results to all populations. Furthermore, although the study provides a predictive model, it requires long-term validation to confirm its accuracy and reliability in clinical settings.

Thus, overall, these articles highlight the promising role of associating radiomics with genomics data as a non-invasive tool in enhancing TC diagnosis and prognosis. However, addressing these limitations in future research will be crucial to enhance the robustness and clinical applicability of the models.

The other papers included in this systematic review examined the combination of radiomic and genomic characteristics in predicting mutation status. Currently, the mutation status is determined through FNA and gene sequencing [54, 55]. Despite its widespread use, FNA can give false-negative [56]. Hence, a non-invasive preoperative screening tool and accurate knowledge of mutation status could be necessary for diagnosis of PTC, providing clinicians novel strategies to optimize therapeutic management.

In Zheng et al.’s work [41], the texture model, utilizing MRI features, demonstrates promising predictive value for BRAF^V600E^ mutation in PTC. Despite that, this study acknowledges limitations, including relatively small sample size, potentially leading to overfitting. Furthermore, the study excluded thyroid lesions smaller than 5 mm and focused only on the largest lesions in patients with multiple PTC.

Also, in the study of Wang and colleagues, US radiomic models based on elastic features and the combination of both the elastic and grayscale features revealed to be a potential tool to assess BRAF^V600E^ mutation status in PTC [42]. Several limitations have been reported in this study, such as: (i) the small sample size, which may lead to selection bias; (ii) radiomic features were extracted from images generated by only two ultrasound devices; (iii) the lack of genetic analysis of the BRAF gene in healthy subjects and, finally, (iv) the absence of external validation data.

On the contrary, in Yoon’s study [43], radiomic features extracted from US did not prove to be reliable for the preoperative diagnosis of the BRAF^V600E^ mutation status in patients with PTC, regardless of tumor size, but only in a subgroup analysis of tumors sized less than 20 mm. Furthermore, the main limitation of this study was related to the inherent observer variability of US, and also that the ROI segmentation was performed by one radiologist, which may have influenced the results. Another limitation may be associated with the enrolled population, which may not accurately reflect the mutation features of the TC population.

In the same context, the performance of a deep learning radiomics nomogram derived from the combination of US radiomics and deep transfer learning (DTL) in preoperative RET/PTC rearrangement detection was investigated [44]. The results showed a valuable predictive ability of the radiomics nomogram and demonstrated that DTL can potentially be integrated into diagnostic procedures to assist clinicians in their medical decisions. However, some limitations have emerged from this work and further studies are needed to validate the predictive ability of the model in a larger sample size and to extend the field to the specific RET rearrangement subtypes.

The predominant molecular alterations observed in PTC are associated with the activation of the MAPK signaling pathway, which includes point mutations in BRAF and RAS genes, and fusions involving the tyrosine kinases RET and NTRK1. Additionally, mutations in PTEN, PIK3CA, and AKT1 genes have been identified with less frequencies [10, 17]. Thus, despite a thorough understanding of the molecular profiles associated with PTCs, the literature lacks studies that utilize radiomic signatures to predict other mutations, such as those in RAS or other key genes, in addition to BRAF and RET. This gap highlights a critical area for future investigation.

Overall, from the analysis of TC radiogenomics literature, a substantial scarcity of work emerges which, although well evaluated by the quality control used in the review, does not allow a homogeneous evaluation and, above all, a comparison of the results.

We could not compare our systematic review with others on this topic because to the best of our knowledge, this is the first review that investigates the potential association of radiomic features with genomic signatures in TC.

On the other hand, other tumor districts, such as colon [57], breast [58–66], prostate [67], lung [68, 69], bladder [70], gliomas [71, 72] of comparable incidence, have favored the production of a large literature. In these cancers quantitative radiomic features associated to the genomic signature were able to reveal tumor heterogeneity, to distinguish molecular subtypes and to unveil novel potentials therapeutic targets. This difference can be possibly, mainly attributable to the imaging methods used in diagnostic practice and the related data made available to the scientific community. In fact, TC is characterized by the prevalent use of US which, due to its intrinsic limitations, does not allow optimal radiomic analysis.

Future research could take advantage of the data deposited in The Cancer Imaging Archive (TCIA). The TCIA, in connection with The Cancer Genome Atlas (TCGA), is a useful tool for radiogenomics providing datasets that combine imaging data with genomic information, helping clinicians in the development of more accurate diagnostic and therapeutic strategies.

### Strengths, limitations and future perspectives

The interplay between radiomic and genomic features plays an important role in the advancement of diagnostic, predictive or prognostic tumor models, representing a promising avenue in cancer research [73]. For this reason, the development of both novel nomograms and genomic methods and their association could be useful for the advancement of personalized medicine.

A major limitation of our work is the low number of studies (*n* = 7) that meet the search criteria on association of radiomics and genomics. Indeed, most of the studies included in the search focus on radiomics underlining the potential of radiomic signatures as non-invasive tool, providing clinicians additional information for preoperative decision-making and prognostic assessment. Notably, the ability to predict lymph node metastasis in PTC using radiomic features from various imaging modalities, such as CT and US, indicates a promising direction for improving patient management strategies.

Another important limitation of this systematic review is certainly the methodological heterogeneity observed among the different studies discussed in the field of radiomics. Indeed, different imaging modalities, such as US, CT and MRI were utilized and this methodological heterogeneity is also evident from genomic methods, where the use of sequencing techniques (i.e., NGS, Sanger sequencing) and PCR introduces an additional layer of variability.

While US is the most used imaging technique as a stand-alone approach to TC, representing the gold standard with FNA for the diagnosis, it represents the less reliable, sonographer-dependent approach compared to other cited modalities. However, considering the widespread and the cost of this imaging technique, more efforts have to be spent to increase its standardization and reproducibility.

Furthermore, the use of different software and methods for generating features complicates the translation of the concept of radiogenomics into clinical practice. In clinical settings, reproducibility and reliability of measures and procedures are crucial. Consequently, the interpretation of results may be influenced by this methodological heterogeneity, highlighting the need for a methodological standardization process.

These characteristics gain more importance if it is considered that the small sample size represents the main common drawback of all examined studies. Finally, although intrinsically heterogeneous, the PTC is the more common TC but not the unique subtype, limiting the generalizability of these findings.

Additionally, one limitation of this systematic review regards the different design of the studies included. Indeed, out of the 7 studies, 1 was prospective, 5 retrospective and 1 was prospective and retrospective multicenter study.

Another limitation of our review could be the bias towards positive findings. Indeed, 6 out of 7 studies included in our analysis state that the association of radiomic and genomic studies is beneficial. Only one study suggests that the association of US-radiomics data and genetic mutations has limited predicting value [43]. The bias towards positive findings could be due to the low number of studies that are available on this topic.

In order to overcome, or mitigate, all these limitations, the use of well-established tools in the bioinformatics field, such as multi-assay experiments, which have recently been demonstrated to be suitable by integrating the radiomics domain [74, 75] should certainly be favored and promoted.

In conclusion, while the synergy between radiomic features and genomic signatures holds promise as a non-invasive tool for enhancing TC diagnosis and management, further researches, with larger cohorts and standardized methodologies, are essential to establish its clinical utility.

## Supplementary Information

Supplementary material 1. PRISMA checklist.

Supplementary material 2. List of studies not included and exclusion reasons.

Supplementary material 3. AMSTAR 2.

Supplementary material 4. Description of radiomic features reported in the studies analyzed.

## Data Availability

All data generated or analyzed during this study are included in this published article and its Additional information files.

## Abbreviations

^131^I: Iodine-131
ARMS RT-PCR: Amplification refractory mutation system real-time polymerase chain reaction
AUC: Area under the curve
CLN: Cervical lymph nodes
CLNM: Cervical lymph nodes metastasis
CT: Computed tomography
DLRN: Deep learning radiomics nomogram
DTL: Deep transfer learning
FNA: Fine-needle aspiration
IHC: Immunohistochemistry
iTRAQ: Isobaric tags for relative and absolute quantitation
LC–MS/MS: Liquid chromatograph-mass spectrometry
LNLNs: Lateral neck lymph nodes
MRI: Magnetic resonance imaging
n: Number
NGS: Next generation sequencing
PCR: Polymerase chain reaction
PET/CT: Positron emission tomography/ computed tomography
PTC: Papillary thyroid cancer
ROI: Region of interest
SPECT/CT: Single photon emission tomography/computed tomography
TC: Thyroid cancer
TCGA: The Cancer Genome Atlas
TCIA: The cancer imaging archive
Tg: Thyroglobulin
TSH: Thyroid-stimulating hormone
US: Ultrasound
WBS: Whole-body hybrid scan
WGCNA: Weighted gene co-expression network analysis
