# Supplementary material for: Association of radiomic features with genomic signatures in thyroid cancer: a systematic review
Source: J Transl Med. 2024 Nov 30;22:1088. doi: 10.1186/s12967-024-05896-z (PMC11608493; doi:10.1186/s12967-024-05896-z)
Supplement: Supplementary file 2 — Supplementary material 2. List of studies not included and exclusion reasons. [file 12967_2024_5896_MOESM2_ESM.pdf]

Additional file 2: List of studies not included and exclusion reasons.

Authors	Year	Title	DOI	Exclusion criteria
Rueff J. *et al.*	2002	DNA polymorphisms as modulators of genotoxicity and cancer	10.1515/BC.2002.099	Review
Mazzanti C. *et al.*	2004	Using gene expression profiling to differentiate benign versus malignant thyroid tumors	10.1158/0008-5472.can-03-3811	No key words
Fujarewicz K. *et al.*	2007	A multi-gene approach to differentiate papillary thyroid carcinoma from benign lesions: gene selection using support vector machines with bootstrapping	10.1677/ERC-06-0048	No key words
Riesco-Eizaguirre G., Santisteban P.	2007	Molecular biology of thyroid cancer initiation	10.1007/s12094-007-0125-1	Review
Guda P. *et al.*	2009	Comparative Analysis of Protein-Protein Interactions in Cancer-Associated Genes	10.1016/S1672-0229(08)60030-3	No key words
Gandhi M. *et al.*	2009	DNA copy number aberrations in thyroid tumors determined using SNP arrays	10.1038/labinvest.2008.137	Conference Abstract
Krause K. *et al.*	2009	Proteomics in thyroid tumor research	10.1210/jc.2009-0308	Review
Carpi A., Nicolini A.	2010	Methodological and clinical progress in thyroid tumor markers	10.1007/s13277-010-0092-y	Conference Abstract
Carpi A. *et al.*	2010	Thyroid tumor marker genomics and proteomics: diagnostic and clinical implications	10.1002/jcp.22187	Review
Silvestri E. *et al.*	2011	Studies of complex biological systems with applications to molecular medicine: The need to integrate transcriptomic and proteomic approaches	10.1155/2011/810242	Review
Cheng I.C. *et al.*	2011	Pleiotropic effects on lung cancer of genetic susceptibility variants identified for other malignancies: The multiethnic cohort	10.1158/1538-7445.AM2011-884	Conference Abstract
Denny J.C. *et al.*	2011	Variants near FOXE1 are associated with hypothyroidism and other thyroid conditions: using electronic medical records for genome- and phenome-wide studies	10.1016/j.ajhg.2011.09.008	No key words
DeNardo G.L, DeNardo S.J.	2012	Concepts, consequences, and implications of theranosis	10.1053/j.semnuclmed.2011.12.003	Review

Hernández-Lemus E., Mejía C.	2012	Inference and analysis of apoptotic pathways in papillary thyroid cancer	N/A	Book chapter
Thomas G. *et al.*	2012	The chernobyl tissue bank - a repository for biomaterial and data used in integrative and systems biology modeling the human response to radiation	10.3390/genes3020278	No key words
Sheng W.-H. *et al.*	2013	Application of proteomics in radiation-related cancer research	10.3760/cma.j.issn.1006-9801.2013.05.026	Review
Katoh M. *et al.*	2013	Cancer genetics and genomics of human FOX family genes	10.1016/j.canlet.2012.09.017	Review
Razzouk S.	2014	Translational genomics and head and neck cancer: Toward precision medicine	10.1111/cge.12487	Review
Crona D.J. *et al.*	2014	A high-throughput cellular genetics approach to identifying genes associated with sorafenib response and toxicity	10.1158/1538-7445.AM2014-5560	Conference Abstract
Bas H. *et al.*	2014	The interplay between thyroid carcinoma cells and immune system leads to macrophages with a chronic inflammatory phenotype	10.1089/thy.2014.2410.abstracts	Conference Abstract
Seifert R. *et al.*	2014	Mutated BRAF V600E in poorly differentiated spindle cell malignancies following the initial diagnosis of melanoma in another site: A diagnostic challenge	10.1093/ajcp/142.suppl1.248	Conference Abstract
Melloni G.E.M. *et al.*	2014	DOTS-Finder: A comprehensive tool for assessing driver genes in cancer genomes	10.1186/gm563	No key words
Weisenberger D.J.	2014	Characterizing DNA methylation alterations from the cancer genome atlas	10.1172/JCI69740	Review
Trarbach E.B. *et al.*	2014	Genome-wide promoter methylation analysis in cytologically indeterminate thyroid nodules	10.1159/000365775	Conference Abstract
Perdichizzi S. *et al.*	2014	Cancer-related genes transcriptionally induced by the fungicide penconazole	10.1016/j.tiv.2013.06.006	No key words
Steward D.L., Kloos R.T.	2014	Clinical diagnostic gene expression thyroid testing	10.1016/j.otc.2014.04.009	Review
Suarez-Kurtz G., Hutz M.H.	2014	Pharmacogenomics in Brazil	10.1016/B978-0-12-386882-4.00045-1	Book chapter

Ozer B, Sezerman O.U.	2015	A novel analysis strategy for integrating methylation and expression data reveals core pathways for thyroid cancer aetiology	10.1186/1471-2164-16-S12-S7	No key words
Gustafson D.L. *et al.*	2015	The flint animal cancer center (FACC) canine tumor cell line panel: A resource for comparative and translational oncology	10.1158/1538-7445.AM2015-5136	Conference Abstract
Yang Z. *et al.*	2015	An integrative pan-cancer-wide analysis of epigenetic enzymes reveals universal patterns of epigenomic deregulation in cancer	10.1186/s13059-015-0699-9	No key words
Stark D. *et al.*	2015	Survival patterns in teenagers and young adults with cancer in the United Kingdom: Comparisons with younger and older age groups	10.1016/j.ejca.2015.08.010	No key words
Liu Q.	2015	Application of metabonomics in early diagnosis of diseases	10.11910/2227-6394.2015.03.02.13	Review
Bann D.V. *et al.*	2015	De novo genome mapping of a follicular variant papillary thyroid cancer	10.1089/thy.2015.29006.abstracts	Conference Abstract
Gordon B.L. *et al.*	2015	Genomic medicine for cancer diagnosis	10.1002/jso.23778	Review
Wojakowska A. *et al.*	2015	Application of metabolomics in thyroid cancer research	10.1155/2015/258763	Review
Voutetakis K. *et al.*	2015	Comparative Meta-Analysis of Transcriptomics Data during Cellular Senescence and In Vivo Tissue Ageing	10.1155/2015/732914	No key words
Rudqvist N. *et al.*	2015	Gene expression signature in mouse thyroid tissue after (131)I and (211)At exposure	10.1186/s13550-015-0137-8	Mice model
Muzza M. *et al.*	2015	Telomerase in differentiated thyroid cancer: promoter mutations, expression and localization	10.1016/j.mce.2014.10.019	No key words
Baskovich B. *et al.*	2016	Expanded genetic screening panel for the Ashkenazi Jewish population	10.1038/gim.2015.123	No key words
Bangi E. *et al.*	2016	A Drosophila approach to personalized cancer therapeutics	10.1158/1557-3265.PMSCLINGEN15-34	Conference Abstract
Riesco-Eizaguirre G., Santisteban P.	2016	Advances in the molecular pathogenesis of thyroid cancer: Lessons from the cancer genome	10.1530/EJE-16-0202	Review

Aherne S.T. *et al.*	2016	Altered expression of mir-222 and mir-25 influences diverse gene expression changes in transformed normal and anaplastic thyroid cells, and impacts on MEK and TRAIL protein expression	10.3892/ijmm.2016.2653	No key words
Lu Z. *et al.*	2016	Clonality analysis of multifocal papillary thyroid carcinoma by using genetic profiles	10.1002/path.4696	No key words
Giordano T.J.	2016	Follicular cell thyroid neoplasia: insights from genomics and The Cancer Genome Atlas research network	10.1097/CCO.0000000000000248	Review
Alaei-Mahabadi B. *et al.*	2016	Global analysis of somatic structural genomic alterations and their impact on gene expression in diverse human cancers	10.1073/pnas.1606220113	No key words
Wei P.J. *et al.*	2016	LNDriver: identifying driver genes by integrating mutation and expression data based on gene-gene interaction network	10.1186/s12859-016-1332-y	No key words
Kirschner L.S. *et al.*	2016	Mouse models of thyroid cancer: A 2015 update	10.1016/j.mce.2015.06.029	Review
Brettschneider J.	2016	Practical uses of quality assessment for high-dimensional gene expression data	10.1007/978-3-319-25454-8_29	Book chapter
Busch E.L. *et al.*	2017	Somatic mutations in CDH1 and CTNNB1 in primary carcinomas at 13 anatomic sites	10.18632/oncotarget.21115	No key words
Fowles J.S. *et al.*	2017	The Flint Animal Cancer Center (FACC) Canine Tumour Cell Line Panel: a resource for veterinary drug discovery, comparative oncology and translational medicine	10.1111/vco.12192	No key words
Chekan M. *et al.*	2017	Molecular profiles of thyroid cancer: Classification based on features of tissue revealed by mas spectrometry imaging	10.1007/s00428-017-2205-0	Conference Abstract
Sollini M. *et al.*	2017	[(18)F]FDG-PET/CT texture analysis in thyroid incidentalomas: preliminary results	10.1186/s41824-017-0009-8	No key words
Zhao Z. *et al.*	2017	[Progress in diagnosis and treatment of radioactive iodine-refractory differentiated thyroid carcinoma]	10.3760/cma.j.issn.1673-0860.2017.12.020	Review
Alsina J. *et al.*	2017	A Concise Atlas of Thyroid Cancer Next-Generation Sequencing Panel ThyroSeq v.2	10.4274/2017.26.suppl.12	Review

Flynn A. *et al.*	2017	Cousins not twins: intratumoural and intertumoural heterogeneity in syndromic neuroendocrine tumours	10.1002/path.4900	No key words
Wei P.-J. *et al.*	2017	Driver finder: A gene length-based network method to identify cancer driver genes	10.1155/2017/4826206	No key words
Imielinski M. *et al.*	2017	Insertions and Deletions Target Lineage-Defining Genes in Human Cancers	10.1016/j.cell.2016.12.025	No key words
Zhang T., Zhang D.	2017	Integrating omics data and protein interaction networks to prioritize driver genes in cancer	10.18632/oncotarget.19481	No key words
Lebeault M. *et al.*	2017	Nationwide French Study of RET Variants Detected from 2003 to 2013 Suggests a Possible Influence of Polymorphisms as Modifiers	10.1089/thy.2016.0399	No key words
Zafon C. *et al.*	2017	Nodular Thyroid Disease and Thyroid Cancer in the Era of Precision Medicine	10.1159/000457793	Review
Vanden Borre P. *et al.*	2017	Pediatric, Adolescent, and Young Adult Thyroid Carcinoma Harbors Frequent and Diverse Targetable Genomic Alterations, Including Kinase Fusions	10.1634/theoncologist.2016-0279	No key words
Sholl L.M. *et al.*	2017	Radiation-associated neoplasia: clinical, pathological and genomic correlates	10.1111/his.13069	Review
Song H. *et al.*	2017	Selective Ablation of Tumor Suppressors in Parafollicular C Cells Elicits Medullary Thyroid Carcinoma	10.1074/jbc.M116.765727	No key words
Thomas G.	2017	Somatic Genomics of Childhood Thyroid Cancer	10.1016/B978-0-12-812768-1.00012-5	Book chapter
Gulec S.	2017	The Art and Science of Thyroid Surgery in the Age of Genomics: 100 years after Theodor Kocher	10.4274/2017.26.suppl.01	Review
Farrokhi Yekta R. *et al.*	2017	The metabolomics and lipidomics window into thyroid cancer research	10.1080/1354750X.2016.1256429	Review

Montero-Conde C. *et al.*	2017	Transposon mutagenesis identifies chromatin modifiers cooperating with Ras in thyroid tumorigenesis and detects ATXN7 as a cancer gene	10.1073/pnas.1702723114	No key words
Luoh S.-W., Flaherty K.T.	2018	When tissue is no longer the issue: Tissue-agnostic cancer therapy comes of age	10.7326/M17-2832	Review
Jastaniah W. *et al.*	2018	Prevalence of hereditary cancer susceptibility syndromes in children with cancer in a highly consanguineous population	10.1016/j.canep.2018.05.006	No key words
Sengupta S. *et al.*	2018	Integrative omics analyses broaden treatment targets in human cancer	10.1186/s13073-018-0564-z	No key words
Zhang Y. *et al.*	2018	Dissecting Pathway Disturbances Using Network Topology and Multi-platform Genomics Data	10.1007/s12561-017-9193-0	No key words
Crezee T. *et al.*	2018	Digoxin treatment for heart disease is associated with a higher tumor differentiation status and favorable clinical outcome in non-medullary thyroid cancer patients	10.1159/000491542	Conference Abstract
Ronsley R. *et al.*	2018	Application of genomics to identify therapeutic targets in recurrent pediatric papillary thyroid carcinoma	10.1101/mcs.a002568	No key words
Fritsche L.G. *et al.*	2018	Association of Polygenic Risk Scores for Multiple Cancers in a Phenome-wide Study: Results from The Michigan Genomics Initiative	10.1016/j.ajhg.2018.04.001	No key words
Liu T. *et al.*	2018	Comparison of the application of B-mode and strain elastography ultrasound in the estimation of lymph node metastasis of papillary thyroid carcinoma based on a radiomics approach	10.1007/s11548-018-1796-5	No key words
Wang Y. *et al.*	2018	Highly Selective 5-Formyluracil Labeling and Genome-wide Mapping Using (2-Benzimidazolyl)Acetonitrile Probe	10.1016/j.isci.2018.10.024	No key words
Sollini M. *et al.*	2018	Texture analysis and machine learning to characterize suspected thyroid nodules and differentiated thyroid cancer: Where do we stand?	10.1016/j.ejrad.2017.12.004	Review

Saini S. *et al.*	2018	Therapeutic advances in anaplastic thyroid cancer: a current perspective	10.1186/s12943-018-0903-0	Review
Dehghannasiri R. *et al.*	2019	Towards precise and cost-effective fusion discovery: A landscape of druggable gene fusions across TCGA cancers	10.1158/1538-7445.SABCS18-2468	Conference Abstract
Mounir M. *et al.*	2019	New functionalities in the TCGAbiolinks package for the study and integration of cancer data from GDC and GTEX	10.1371/journal.pcbi.1006701	No key words
Farina L., Filetti S.	2019	A network approach to investigate the impact of genetic modifiers on driver-guided carcinogenesis	10.1089/sysm.2019.29005	Conference Abstract
Huang Y. *et al.*	2019	Comparison of ultrasound radiomics with conventional imaging models: diagnosis of central cervical lymph node metastasis in papillary thyroid carcinoma	10.3760/cma.j.issn.1004-4477.2019.10.011	No english
Wang W., Li Y.	2019	[Radiomics for prediction of central lymph node metastasis in the neck in patients with thyroid papillary carcinoma]	10.12122/j.issn.1673-4254.2019.09.15	No english
Becker P.S. *et al.*	2019	A Multi-Omic Precision Medicine Clinical Trial in Acute Leukemia	10.1182/blood-2019-130996	Conference Abstract
Hao Y. *et al.*	2019	Analytical Verification Performance of Afirma Genomic Sequencing Classifier in the Diagnosis of Cytologically Indeterminate Thyroid Nodules	10.3389/fendo.2019.00438	No key words
Park V.Y. *et al.*	2019	Association Between Radiomics Signature and Disease-Free Survival in Conventional Papillary Thyroid Carcinoma	10.1038/s41598-018-37748-4	No key words
Govindan R., Devarakonda S.	2019	Cancer Genomics for the Clinician	10.1891/9780826168689	Book chapter
Dragon J. *et al.*	2019	Comparison of RNA sequencing data generated from formalin-fixed, paraffin-embedded (FFPE) papillary thyroid carcinoma samples using different library preparation methods	10.1158/1538-7445.SABCS18-1664	Conference Abstract
Chen B. *et al.*	2019	Computed Tomography Radiomic Nomogram for Preoperative Prediction of Extrathyroidal Extension in Papillary Thyroid Carcinoma	10.3389/fonc.2019.00829	No key words

Wells J.D., Miller T.W.	2019	Development of pan-cancer transcriptional signatures that predict chemosensitivity	10.1158/1538-7445.SABCS18-4239	Conference Abstract
Zafon C. *et al.*	2019	DNA methylation in thyroid cancer	10.1530/ERC-19-0093	Review
Ali S.Z. *et al.*	2019	Extending expressed RNA genomics from surgical decision making for cytologically indeterminate thyroid nodules to targeting therapies for metastatic thyroid cancer	10.1002/cncy.22132	No key words
Mohyuddin A. *et al.*	2019	Gallic acid functionalized UiO-66 for the recovery of ribosylated metabolites from human urine samples	10.1016/j.talanta.2019.03.072	No key words
Ciampi R. *et al.*	2019	Genetic Landscape of Somatic Mutations in a Large Cohort of Sporadic Medullary Thyroid Carcinomas Studied by Next-Generation Targeted Sequencing	10.1016/j.isci.2019.09.030	No key words
Cavadas B. *et al.*	2019	Genomic and transcriptomic characterization of the mitochondrial-rich oncocytic phenotype on a thyroid carcinoma background	10.1016/j.mito.2018.04.001	No key words
Du G. *et al.*	2019	High Expression of miR-206 Predicts Adverse Outcomes: A Potential Therapeutic Target for Esophageal Cancer	10.2174/1386207322666191018145825	No key words
Yu X. *et al.*	2019	Key candidate genes associated with BRAF(V600E) in papillary thyroid carcinoma on microarray analysis	10.1002/jcp.28906	No key words
Boufraqech M., Nilubol N.	2019	Multi-omics Signatures and Translational Potential to Improve Thyroid Cancer Patient Outcome	10.3390/cancers11121988	Review
Hossain M.A. *et al.*	2019	Network-based approach to identify key candidate genes and pathways shared by thyroid cancer and chronic kidney disease	10.1016/j.imu.2019.100240	No key words
Papathomas T.G., Nosé V.	2019	New and Emerging Biomarkers in Endocrine Pathology	10.1097/PAP.0000000000000227	Review
Caso R. *et al.*	2019	Papillary thyroid carcinoma with bilateral axillary lymph node involvement: a case report outlining hypothesis for locoregional spread and therapeutic implications	10.21037/acr.2019.01.02	No key words

Bacolod M.D. *et al.*	2019	Pathways- and epigenetic-based assessment of relative immune infiltration in various types of solid tumors	10.1016/bs.acr.2019.01.003	Book chapter
Liu T. *et al.*	2019	Prediction of Lymph Node Metastasis in Patients With Papillary Thyroid Carcinoma: A Radiomics Method Based on Preoperative Ultrasound Images	10.1177/1533033819831713	No key words
Lu W. *et al.*	2019	Radiomic analysis for preoperative prediction of cervical lymph node metastasis in patients with papillary thyroid carcinoma	10.1016/j.ejrad.2019.07.018	No key words
Acuña-Ruiz A. *et al.*	2019	SIX1 is overexpressed in anaplastic thyroid cancer and promotes epithelial-to-mesenchymal transition	10.1159/000501012	Conference Abstract
Shi Z. *et al.*	2019	Systematic evaluation of cancer-specific genetic risk score for 11 types of cancer in The Cancer Genome Atlas and Electronic Medical Records and Genomics cohorts	10.1002/cam4.2143	No key words
Shaha A.R., Tuttle R.M.	2019	Thyroid cancer staging and genomics	10.21037/atm.2019.03.11	Editorial
Zhou S.-C. *et al.*	2019	Ultrasound radimoics nomogram for predicting lymph node metastasis in papillary thyroid carcinoma	10.1016/j.ultrasmedbio.2019.07.673	Conference Abstract
Li C. *et al.*	2020	Identification of microrna-related tumorigenesis variants and genes in the cancer genome atlas (Tcga) data	10.3390/genes11090953	No key words
Huang X. *et al.*	2020	Genomic analysis of the prognostic value of colony-stimulating factors (CSFs) and colony-stimulating factor receptors (CSFRs) across 24 solid cancer types	10.21037/atm-20-5363	No key words
Li Q.-T. *et al.*	2020	Integrative analysis of Siglec-15 mRNA in human cancers based on data mining	10.7150/jca.38747	No key words
Sun Q. *et al.*	2020	Computational Identification of Tumor Suppressor Genes Based on Gene Expression Profiles in Normal and Cancerous Gastrointestinal Tissues	10.1155/2020/2503790	No key words
Gao R. *et al.*	2020	Inferring copy number substructurefrom single-cell transcriptomics in human tumors withCopyKat	10.1158/1538-7445.TUMHET2020-PO-020	Conference Abstract

LaBarge B. *et al.*	2020	27. Genome imaging of head and neck solid tumors: Oropharyngeal, tongue, and thyroid cancers	10.1016/j.cancergen.2020.04.031	Conference Abstract
Cumbo F. *et al.*	2020	A brain-inspired hyperdimensional computing approach for classifying massive DNA methylation data of cancer	10.3390/A13090233	No key words
Shao X. *et al.*	2020	A Clinical Genomics-Guided Prioritizing Strategy Enables Selecting Proper Cancer Cell Lines for Biomedical Research	10.1016/j.isci.2020.101748	No key words
Zhao Y. *et al.*	2020	A Germline CHEK2 Mutation in a Family with Papillary Thyroid Cancer	10.1089/thy.2019.0774	No key words
García-Vence M. *et al.*	2020	A Novel Nanoproteomic Approach for the Identification of Molecular Targets Associated with Thyroid Tumors	10.3390/nano10122370	No key words
Ehrhardt J.D. Jr, Güleç S.	2020	A Review of the History of Radioactive Iodine Theranostics: The Origin of Nuclear Ontology	10.4274/mirt.galenos.2020.83703	Review
Zhou S.C. *et al.*	2020	An Ultrasound Radiomics Nomogram for Preoperative Prediction of Central Neck Lymph Node Metastasis in Papillary Thyroid Carcinoma	10.3389/fonc.2020.01591	No key words
Guo B.J. *et al.*	2020	Benign and malignant thyroid classification using computed tomography radiomics	10.1117/12.2549087	No free article
Sahajpal N.S. *et al.*	2020	Clinical utility of comprehensive genomic pathway and integrated network analyses in personalized oncology	10.1200/JCO.2020.38.15_suppl.e14051	Conference Abstract
Weeraratne D. *et al.*	2020	Comprehensive analysis of advanced-stage solid tumors from TCGA reveal widespread variation of genomics evidence levels across cancer types	10.1200/JCO.2020.38.15_suppl.e13547	Conference Abstract
Shin M.H. *et al.*	2020	Current Insights into Combination Therapies with MAPK Inhibitors and Immune Checkpoint Blockade	10.3390/ijms21072531	Review
Hong S. *et al.*	2020	Development of circulating free DNA methylation markers for thyroid nodule diagnostics	10.1016/j.annonc.2020.10.301	Conference Abstract

Hong S. *et al*.	2020	Discovery and validation of novel DNA methylation markers for thyroid nodule diagnostics in plasma	10.1016/j.annonc.2020.08.104	Conference Abstract
Barletta-Carrillo C. *et al.*	2020	Evaluation of germline RET proto-oncogene variants in Peruvian patients with medullary thyroid carcinoma	10.24875/HGMX.20000050	No key words
Porosnicu M. *et al.*	2020	Exceptional Responders to Immunotherapy in Head and Neck Cancer	10.1016/j.ijrobp.2019.11.358	Conference Abstract
Bhalla S. *et al.*	2020	Expression based biomarkers and models to classify early and late-stage samples of Papillary Thyroid Carcinoma	10.1371/journal.pone.0231629	No key words
Qu N. *et al.*	2020	Genomic and Transcriptomic Characterization of Sporadic Medullary Thyroid Carcinoma	10.1089/thy.2019.0531	No key words
Rao S.N., Bernet V.	2020	Indeterminate thyroid nodules in the era of molecular genomics	10.1002/mgg3.1288	Review
Guo L. *et al.*	2020	Integrative omics analysis reveals relationships of genes with synthetic lethal interactions through a pan-cancer analysis	10.1016/j.csbj.2020.10.015	No key words
Bangaraiahgari R. *et al.*	2020	Is there adenoma-carcinoma sequence between benign adenoma and papillary cancer of thyroid: A genomic linkage study	10.1016/j.amsu.2020.11.069	No key words
Yu J. *et al.*	2020	Lymph node metastasis prediction of papillary thyroid carcinoma based on transfer learning radiomics	10.1038/s41467-020-18497-3	No key words
Wang H. *et al.*	2020	Machine learning-based multiparametric MRI radiomics for predicting the aggressiveness of papillary thyroid carcinoma	10.1016/j.ejrad.2019.108755	No key words
Akyay O.Z. *et al.*	2020	Mapping the Molecular Basis and Markers of Papillary Thyroid Carcinoma Progression and Metastasis Using Global Transcriptome and microRNA Profiling	10.1089/omi.2019.0188	No key words
Song Y.S., Park Y.J.	2020	Mechanisms of TERT Reactivation and Its Interaction with BRAFV600E	10.3803/EnM.2020.304	Review
Jikuzono T. *et al.*	2020	Microarray analysis of formalin-fixed, paraffin-embedded follicular thyroid carcinoma samples from patients who developed postoperative distant metastasis	10.1186/s13104-020-05080-8	No key words

Nylén C. *et al.*	2020	Molecular Markers Guiding Thyroid Cancer Management	10.3390/cancers12082164	Review
Pozdeyev N. *et al.*	2020	Molecular therapeutics for anaplastic thyroid cancer	10.1016/j.semcancer.2020.01.005	No key words
Piga I. *et al.*	2020	Molecular trait of follicular-patterned thyroid neoplasms defined by MALDI-imaging	10.1016/j.bbapap.2020.140511	No key words
Jin Y. *et al.*	2020	Mouse models of thyroid cancer: Bridging pathogenesis and novel therapeutics	10.1016/j.canlet.2019.09.017	Review
Hu W. *et al.*	2020	MRI-based radiomics analysis to predict preoperative lymph node metastasis in papillary thyroid carcinoma	10.21037/gs-20-479	No key words
Jiang M. *et al.*	2020	Nomogram Based on Shear-Wave Elastography Radiomics Can Improve Preoperative Cervical Lymph Node Staging for Papillary Thyroid Carcinoma	10.1089/thy.2019.0780	No key words
Iacobas D.A.	2020	Powerful quantifiers for cancer transcriptomics	10.5306/wjco.v11.i9.679	Review
Zhang H. *et al.*	2020	Prediction of Cervical Lymph Node Metastasis Using MRI Radiomics Approach in Papillary Thyroid Carcinoma: A Feasibility Study	10.1177/1533033820969451	No key words
Zhou Y. *et al.*	2020	Radiomics analysis of dual-energy CT-derived iodine maps for diagnosing metastatic cervical lymph nodes in patients with papillary thyroid cancer	10.1007/s00330-020-06866-x	No key words
Kwon M.R. *et al.*	2020	Radiomics Based on Thyroid Ultrasound Can Predict Distant Metastasis of Follicular Thyroid Carcinoma	10.3390/jcm9072156	No key words
Park V.Y. *et al.*	2020	Radiomics signature for prediction of lateral lymph node metastasis in conventional papillary thyroid carcinoma	10.1371/journal.pone.0227315	No key words
Kwon M.R. *et al.*	2020	Radiomics Study of Thyroid Ultrasound for Predicting BRAF Mutation in Papillary Thyroid Carcinoma: Preliminary Results	10.3174/ajnr.A6505	No key words
Mosele F. *et al.*	2020	Recommendations for the use of next-generation sequencing (NGS) for patients with metastatic cancers: a report from the ESMO Precision Medicine Working Group	10.1016/j.annonc.2020.07.014	Review

Pan Z. *et al.*	2020	The differences of regulatory networks between papillary and anaplastic thyroid carcinoma: an integrative transcriptomics study	10.1080/15384047.2020.1803009	No key words
Coelho M. *et al.*.	2020	The Potential of Metabolomics in the Diagnosis of Thyroid Cancer	10.3390/ijms21155272	Review
Sajeev V. *et al.*.	2020	Thyroid cancer prediction using gene expression profile, pharmacogenomic variants and quantum image processing in deep learning platform-a theranostic approach	10.1109/INCET49848.2020.9154041	Conference paper
Donnelly M.K.	2020	Thyroid Cancer: Implications of Genomics for Care and Practice	10.1188/20.CJON.483-487	Review
Li F. *et al.*	2020	Using ultrasound features and radiomics analysis to predict lymph node metastasis in patients with thyroid cancer	10.1186/s12893-020-00974-7	No key words
Puxeddu E. *et al.*	2020	What Is New in Thyroid Cancer: The Special Issue of the Journal Cancers	10.3390/cancers12103036	Editorial
Zhao C.-K. *et al.*	2021	A Comparative Analysis of Two Machine Learning-Based Diagnostic Patterns with Thyroid Imaging Reporting and Data System for Thyroid Nodules: Diagnostic Performance and Unnecessary Biopsy Rate	10.1089/thy.2020.0305	No key words
He J. *et al.*	2021	A pilot study of radiomics signature based on biparametric MRI for preoperative prediction of extrathyroidal extension in papillary thyroid carcinoma	10.3233/XST-200760	No key words
Wang X. *et al.*	2021	A Radiomic Nomogram for the Ultrasound-Based Evaluation of Extrathyroidal Extension in Papillary Thyroid Carcinoma	10.3389/fonc.2021.625646	No key words
Li Y.Y. *et al.*	2021	A Thyroid Ultrasound Image-based Artificial Intelligence Model for Diagnosis of Central Compartment Lymph Node Metastasis in Papillary Thyroid Carcinoma	10.3881/j.issn.1000-503X.13823	No english
Chatsirisupachai K. *et al.*	2021	An integrative analysis of the age-associated multi-omic landscape across cancers	10.1038/s41467-021-22560-y	No key words
Abe I, Lam A.K.	2021	Anaplastic Thyroid Carcinoma: Current Issues in Genomics and Therapeutics	10.1007/s11912-021-01019-9	No key words

Xiong Y. *et al.*	2021	Application of biomarkers in the diagnosis of uncertain samples of core needle biopsy of thyroid nodules	10.1007/s00428-021-03161-y	No key words
Bagante F. *et al.*	2021	Artificial neural networks for multi-omics classifications of hepato-pancreato-biliary cancers: towards the clinical application of genetic data	10.1016/j.ejca.2021.01.049	No key words
Sanghi A. *et al.*	2021	Chromatin accessibility associates with protein-RNA correlation in human cancer	10.1038/s41467-021-25872-1	No key words
Li J. *et al.*	2021	Computed Tomography-Based Radiomics Model to Predict Central Cervical Lymph Node Metastases in Papillary Thyroid Carcinoma: A Multicenter Study	10.3389/fendo.2021.741698	No key words
Fussey J.M. *et al.*	2021	Diagnostic RET genetic testing in 1,058 index patients: A UK centre perspective	10.1111/cen.14395	No key words
Gul M. *et al.*	2021	Diagnostic Utility of Radiomics in Thyroid and Head and Neck Cancers	10.3389/fonc.2021.639326	Review
Vasaikar S.V. *et al.*	2021	EMTome: a resource for pan-cancer analysis of epithelial-mesenchymal transition genes and signatures	10.1038/s41416-020-01178-9	No key words
Janovitz T. *et al.*	2021	Genomic profile of columnar cell variant of papillary thyroid carcinoma	10.1111/his.14374	No key words
Barletta J.A. *et al.*	2021	Genomics and Epigenomics of Medullary Thyroid Carcinoma: From Sporadic Disease to Familial Manifestations	10.1007/s12022-021-09664-3	Review
N/A	2021	Genomics of Chernobyl Cancers Revealed	10.1158/2159-8290.CD-NB2021-0346	Conference Abstract
Dong X. *et al.*	2021	Identification and validation of L Antigen Family Member 3 as an immune-related biomarker associated with the progression of papillary thyroid cancer	10.1016/j.intimp.2020.107267	No key words
Ren H. *et al.*	2021	Identification of a Six Gene Prognosis Signature for Papillary Thyroid Cancer Using Multi-Omics Methods and Bioinformatics Analysis	10.3389/fonc.2021.624421	No key words

Almansoori A. *et al.*	2021	Identifying Diagnostic and Prognostic targets for Papillary Thyroid Carcinoma through mining Gene Expression BIG Datasets using Adaptive Filtering and Advanced Bioinformatics Algorithms	10.1109/DESE54285.2021.9719384	Conference paper
Potter S.L. *et al.*	2021	Integrated DNA and RNA sequencing reveals targetable alterations in metastatic pediatric papillary thyroid carcinoma	10.1002/pbc.28741	No key words
Jia Z. *et al.*	2021	Integration of transcriptomics and metabolomics reveals anlotinib-induced cytotoxicity in colon cancer cells	10.1016/j.gene.2021.145625	No key words
Hu S.-P. *et al.*	2021	LncRNA HCP5 as a potential therapeutic target and prognostic biomarker for various cancers: a meta-analysis and bioinformatics analysis	10.1186/s12935-021-02404-x	Review
Qin H. *et al.*	2021	Magnetic resonance imaging (MRI) radiomics of papillary thyroid cancer (PTC): a comparison of predictive performance of multiple classifiers modeling to identify cervical lymph node metastases before surgery	10.1007/s11547-021-01393-1	No key words
Miller K.C., Chintakuntlawar A.V.	2021	Molecular-Driven Therapy in Advanced Thyroid Cancer	10.1007/s11864-021-00822-7	Review
Lu J. *et al.*	2021	Multi-Omics Analysis of Fatty Acid Metabolism in Thyroid Carcinoma	10.3389/fonc.2021.737127	No key words
Bi G. *et al.*	2021	Multi-omics characterization and validation of invasiveness-related molecular features across multiple cancer types	10.1186/s12967-021-02773-x	No key words
Tung C.-B. *et al.*	2021	Multi-omics reveal the immunological role and the theragnostic value of miR-216a/GDF15 axis in human colon adenocarcinoma	10.3390/ijms222413636	No key words
Edwards K. *et al.*	2021	Multiparametric Radiomics for Predicting the Aggressiveness of Papillary Thyroid Carcinoma Using Hyperspectral Images	10.1117/12.2582147	No key words
Rabold K. *et al.*	2021	Myeloid cell programming in non-medullary thyroid carcinoma	10.1089/thy.2021.29115.abstracts	Conference Abstract
Naglah A. *et al.*.	2021	Novel MRI-Based CAD System for Early Detection of Thyroid Cancer Using Multi-Input CNN	10.3390/s21113878	No key words

Liu Y. *et al.*	2021	Omics- and Pharmacogenomic Evidence for the Prognostic, Regulatory, and Immune-Related Roles of PBK in a Pan-Cancer Cohort	10.3389/fmolb.2021.785370	No key words
Liu P.	2021	Pan-Cancer DNA Methylation Analysis and Tumor Origin Identification of Carcinoma of Unknown Primary Site Based on Multi-Omics	10.3389/fgene.2021.798748	No key words
Zheng X. *et al.*	2021	Pan-cancer evaluation of gene expression and somatic alteration data for cancer prognosis prediction	10.1186/s12885-021-08796-3	No key words
Prete A. *et al.*	2021	Poorly Differentiated and Anaplastic Thyroid Cancer: Insights into Genomics, Microenvironment and New Drugs	10.3390/cancers13133200	Review
Chen Y. *et al.*	2021	Predictions for central lymph node metastasis of papillary thyroid carcinoma via CNN-based fusion modeling of ultrasound images	10.18280/ts.380310	No key words
Ceriani L. *et al.*	2021	Radiomics Analysis of [(18)F]-Fluorodeoxyglucose-Avid Thyroid Incidentalomas Improves Risk Stratification and Selection for Clinical Assessment	10.1089/thy.2020.0224	No key words
Zhou Y. *et al.*	2021	Radiomics based on arterial-venous mixed images derived from dual-energy CT data in diagnosis of lymph nodes metastasis of papillary thyroid cancer	10.3760/cma.j.cn112149-20210421-00395	No english
Wei R. *et al.*	2021	Radiomics based on multiparametric MRI for extrathyroidal extension feature prediction in papillary thyroid cancer	10.1186/s12880-021-00553-z	No key words
Cao Y. *et al.*	2021	Radiomics in Differentiated Thyroid Cancer and Nodules: Explorations, Application, and Limitations	10.3390/cancers13102436	Review
Corino V. *et al.*	2021	Radiomics-based prediction of response to multikinase inhibitors in radioiodine-refractory differentiated thyroid cancer patients	10.1200/JCO.2021.39.15_suppl.6077	Conference Abstract
Cheng Z. *et al.*	2021	Selenite Induces Cell Cycle Arrest and Apoptosis via Reactive Oxygen Species-Dependent Inhibition of the AKT/mTOR Pathway in Thyroid Cancer	10.3389/fonc.2021.668424	No key words

Yan T. *et al.*	2021	Single-Cell Transcriptomic Analysis of Ecosystems in Papillary Thyroid Carcinoma Progression	10.3389/fendo.2021.729565	No key words
Thomas G.A.	2021	The Chernobyl Tissue Bank-a resource for radiation research	10.1007/s12551-021-00844-3	Conference Abstract
Singh A. *et al.*	2021	The Genomic Landscape of Thyroid Cancer Tumourigenesis and Implications for Immunotherapy	10.3390/cells10051082	Review
Stauffer E. *et al.*	2021	Transcriptomic landscape of radiation-induced murine thyroid proliferative lesions	10.1530/ERC-21-0019	No key words
Tong Y. *et al.*	2021	Ultrasound-Based Radiomic Nomogram for Predicting Lateral Cervical Lymph Node Metastasis in Papillary Thyroid Carcinoma	10.1016/j.acra.2020.07.017	No key words
Morelli E. *et al.*	2021	Ntrk gene rearrangements in niftp: Which role?	N/A	Conference Abstract
Su H. *et al.*	2022	A comprehensive investigation on pan-cancer impacts of constitutive centromere associated network gene family by integrating multi-omics data: A CONSORT-compliant article	10.1097/MD.0000000000028821	No key words
Lai L. *et al.*	2022	A computed tomography-based radiomic nomogram for predicting lymph node metastasis in patients with early-stage papillary thyroid carcinoma	10.1177/02841851211054194	No key words
Zhou M. *et al.*	2022	A pan-cancer analysis of the expression of STAT family genes in tumors and their relationship to the tumor microenvironment	10.3389/fonc.2022.925537	No key words
Wen Q. *et al.*	2022	A radiomics nomogram for the ultrasound-based evaluation of central cervical lymph node metastasis in papillary thyroid carcinoma	10.3389/fendo.2022.1064434	No key words
Naglah A. *et al.*	2022	A review of texture-centric diagnostic models for thyroid cancer using convolutional neural networks and visualized texture patterns	10.1016/B978-0-12-819872-8.00019-7	Review
Lu W.W. *et al.*	2022	A Review of the Role of Ultrasound Radiomics and Its Application and Limitations in the Investigation of Thyroid Disease	10.12659/MSM.937738	Review

Kim J.T. *et al.*	2022	Adrenomedullin2 stimulates progression of thyroid cancer in mice and humans under nutrient excess conditions	10.1002/path.5997	No key words
Paul R. *et al.*	2022	An Artificial Intelligence Ultrasound Platform for Screening and Staging of Thyroid Cancer	10.1016/j.ijrobp.2021.12.023	Conference Abstract
Paul R. *et al.*	2022	An Artificial Intelligence Ultrasound Platform for Screening and Staging Thyroid C	10.1016/j.ijrobp.2022.07.1404	Conference Abstract
Lu B. *et al.*	2022	An ultrasound model for predicting recurrence of papillary thyroid carcinoma after complete endoscopic resection	10.5114/wiitm.2022.116419	No key words
Sorrenti S. *et al.*	2022	Artificial Intelligence for Thyroid Nodule Characterization: Where Are We Standing?	10.3390/cancers14143357	Review
Zhu W. *et al.*	2022	Artificial Neural Network-Based Ultrasound Radiomics Can Predict Large-Volume Lymph Node Metastasis in Clinical N0 Papillary Thyroid Carcinoma Patients	10.1155/2022/7133972	No key words
Klubo-Gwiezdzinska J.	2022	Childhood Exposure to Excess Ionizing Radiation Is Associated with Dose-Dependent Fusions as Molecular Drivers of Papillary Thyroid Cancer	10.1089/ct.2022;34.161-164	No key words
Zhang C. *et al.*	2022	Classification of Thyroid Nodules by Using Deep Learning Radiomics Based on Ultrasound Dynamic Video	10.1002/jum.16006	No key words
Ragusa F. *et al.*	2022	Combination Strategies Involving Immune Checkpoint Inhibitors and Tyrosine Kinase or BRAF Inhibitors in Aggressive Thyroid Cancer	10.3390/ijms23105731	Review
Wang Y. *et al.*	2022	Comparing Bayesian-Based Reconstruction Strategies in Topology-Based Pathway Enrichment Analysis	10.3390/biom12070906	No key words
Dondi F. *et al.*	2022	Comparison between Two Different Scanners for the Evaluation of the Role of (18)F-FDG PET/CT Semiquantitative Parameters and Radiomics Features in the Prediction of Final Diagnosis of Thyroid Incidentalomas	10.3390/jcm11030615	No key words

Zhang C. *et al.*	2022	Construction of a Diagnostic Model for Lymph Node Metastasis of the Papillary Thyroid Carcinoma Using Preoperative Ultrasound Features and Imaging Omics	10.1155/2022/1872412	No key words
Li Z. *et al.*	2022	Contrast-Enhanced CT-Based Radiomics for the Differentiation of Nodular Goiter from Papillary Thyroid Carcinoma in Thyroid Nodules	10.2147/CMAR.S353877	No key words
Pereira B. *et al.*	2022	Contribution and clinical relevance of germline variation to the cancer transcriptome	10.1186/s12885-022-09757-0	No key words
Ali S. *et al.*	2022	Decoding Cancer Immunoediting of Tumor Microenvironment Provides Immunogenomic Marker for Thyroid Cancer Screening	10.1210/jendso/bvac150.1645	Conference Abstract
Leitch K. *et al.*	2022	Detecting Aggressive Papillary Thyroid Carcinoma Using Hyperspectral Imaging and Radiomic Features	10.1117/12.2611842	No key words
Ju S.H. *et al.*	2022	Development of Metabolic Synthetic Lethality and Its Implications for Thyroid Cancer	10.3803/EnM.2022.1402	No key words
Kim Y.H. *et al.*	2022	DIFFERENT METABOLIC PHENOTYPES IN THYROID CANCERS ACCORDING TO DRIVER MUTATIONS	10.1089/thy.2022.29138.abstracts	Conference Abstract
Liu K. *et al.*	2022	Differentiation of predominantly osteolytic from osteoblastic spinal metastases based on standard magnetic resonance imaging sequences: a comparison of radiomics model versus semantic features logistic regression model findings	10.21037/qims-22-267	No key words
Agyekum E.A. *et al.*	2022	Evaluation of Cervical Lymph Node Metastasis in Papillary Thyroid Carcinoma Using Clinical-Ultrasound Radiomic Machine Learning-Based Model	10.3390/cancers14215266	No key words
Kong D. *et al.*.	2022	Evaluation of Radiomics Models Based on Computed Tomography for Distinguishing Between Benign and Malignant Thyroid Nodules	10.1097/RCT.0000000000001352	No key words
Yu P. *et al.*	2022	Extrathyroidal Extension Prediction of Papillary Thyroid Cancer With Computed Tomography Based Radiomics Nomogram: A Multicenter Study	10.3389/fendo.2022.874396	No key words

Rice S.V. *et al.*	2022	Fuzzion2: Fast, sensitive detection of known gene fusions by fuzzy pattern matching for clinical testing and large-scale data mining	10.1158/1538-7445.AM2022-4092	Conference Abstract
Zavaleta E. *et al.*	2022	Genetic Characterization in High-Risk Individuals from a Low-Resource City of Peru	10.3390/cancers14225603	No key words
Melone V. *et al.*	2022	Identification of functional pathways and molecular signatures in neuroendocrine neoplasms by multi-omics analysis	10.1186/s12967-022-03511-7	No key words
Fan X. *et al.*	2022	Identification of immune-related ferroptosis prognostic marker and in-depth bioinformatics exploration of multi-omics mechanisms in thyroid cancer	10.3389/fmolb.2022.961450	No key words
Almansoori A. *et al.*	2022	In silico Analysis of Publicly Available Transcriptomics Data Identifies Putative Prognostic and Therapeutic Molecular Targets for Papillary Thyroid Carcinoma	10.2147/IJGM.S345336	No key words
Zaballos M.A. *et al.*	2022	Inhibiting ERK dimerization ameliorates BRAF-driven anaplastic thyroid cancer	10.1007/s00018-022-04530-9	No key words
Shi X. *et al.*	2022	Integrated proteogenomic characterization of medullary thyroid carcinoma	10.1038/s41421-022-00479-y	No key words
Zhao Y. *et al.*	2022	Integrative analysis deciphers the heterogeneity of cancer-associated fibroblast and implications on clinical outcomes in ovarian cancers	10.1016/j.csbj.2022.11.025	No key words
Yao Y. *et al.*	2022	Integrative Analysis of DNA Methylation and Gene Expression Identified Follicular Thyroid Cancer-Specific Diagnostic Biomarkers	10.3389/fendo.2021.736068	No key words
Xu X.Q. *et al.*	2022	Iodine Maps from Dual-Energy CT to Predict Extrathyroidal Extension and Recurrence in Papillary Thyroid Cancer Based on a Radiomics Approach	10.3174/ajnr.A7484	No key words
Minna E. *et al.*	2022	Medullary Thyroid Carcinoma Mutational Spectrum Update and Signaling-Type Inference by Transcriptional Profiles: Literature Meta-Analysis and Study of Tumor Samples	10.3390/cancers14081951	Meta-analysis

Pasculli A. *et al.*	2022	Metabolomics and thyroid cancer: a pioneering field	10.1111/eci.13796	Conference Abstract
Kim G.H. *et al.*	2022	Multi-omics analysis revealed TEK and AXIN2 are potential biomarkers in multifocal papillary thyroid cancer	10.1186/s12935-022-02606-x	No key words
Dai Z. *et al.*	2022	Multimodality MRI-based radiomics for aggressiveness prediction in papillary thyroid cancer	10.1186/s12880-022-00779-5	No key words
Li H. *et al.*	2022	Nav1.6 promotes the progression of human follicular thyroid carcinoma cells via JAK-STAT signaling pathway	10.1016/j.prp.2022.153984	No key words
Li F. *et al.*	2022	NESM: a network embedding method for tumor stratification by integrating multi-omics data	10.1093/g3journal/jkac243	No key words
Lieberman L., Worden F.	2022	Novel Therapeutics for Advanced Differentiated Thyroid Cancer	10.1016/j.ecl.2021.11.019	Review
Wang X. *et al.*	2022	Pan-Cancer Analysis Reveals Genomic and Clinical Characteristics of TRPV Channel-Related Genes	10.3389/fonc.2022.813100	No key words
Lam A.K.	2022	Papillary Thyroid Carcinoma: Current Position in Epidemiology, Genomics, and Classification	10.1007/978-1-0716-2505-7_1	Book chapter
Jiang Z.	2022	Prediction of BRAF gene mutation in thyroid papillary carcinoma based on 18F-FDG-PET imaging radiomics model	10.1007/s00259-022-05924-4	Conference Abstract
de Koster E.J. *et al.*	2022	Quantitative classification and radiomics of [(18)F]FDG-PET/CT in indeterminate thyroid nodules	10.1007/s00259-022-05712-0	No key words
Giovanella L. *et al.*	2022	Radiomics analysis improves (18)FDG PET/CT-based risk stratification of cytologically indeterminate thyroid nodules	10.1007/s12020-021-02856-1	No key words
Wu X. *et al.*	2022	Radiomics Analysis of Computed Tomography for Prediction of Thyroid Capsule Invasion in Papillary Thyroid Carcinoma: A Multi-Classifier and Two-Center Study	10.3389/fendo.2022.849065	No key words
Lu W.J. *et al.*	2022	RADIOMICS BASED ON TWO-DIMENSIONAL AND THREE-DIMENSIONAL ULTRASOUND FOR EXTRATHYROIDAL EXTENSION FEATURE PREDICTION IN PAPILLARY THYROID CARCINOMA	10.4183/aeb.2022.407	No key words

Zhao L., Ma B.	2022	Radiomics Features of Different Sizes of Medullary Thyroid Carcinoma (MTC) and Papillary Thyroid Carcinoma (PTC) Tumors: A Comparative Study	10.1177/11795549221097675	No key words
Zhou Y. *et al.*	2022	Radiomics from Primary Tumor on Dual-Energy CT Derived Iodine Maps can Predict Cervical Lymph Node Metastasis in Papillary Thyroid Cancer	10.1016/j.acra.2021.06.014	No key words
Yang G. *et al.*	2022	Radiomics Profiling Identifies the Value of CT Features for the Preoperative Evaluation of Lymph Node Metastasis in Papillary Thyroid Carcinoma	10.3390/diagnostics12051119	No key words
Brozou T. *et al.*.	2022	RECURRENT PATHOGENIC GERMLINE CHEK2 VARIANTS IN PEDIATRIC PATIENTS WITH HEMATOLOGICAL MALIGNANCIES	10.1097/01.HS9.0000852292.38263.b8	Conference Abstract
Rabold K. *et al.*	2022	Reprogramming of myeloid cells and their progenitors in patients with non-medullary thyroid carcinoma	10.1038/s41467-022-33907-4	No key words
Zhu N. *et al.*	2022	REV1: A novel biomarker and potential therapeutic target for various cancers	10.3389/fgene.2022.997970	No key words
Chen Z. *et al.*	2022	Single-Cell RNA Sequencing Revealed a 3-Gene Panel Predicted the Diagnosis and Prognosis of Thyroid Papillary Carcinoma and Associated With Tumor Immune Microenvironment	10.3389/fonc.2022.862313	No key words
Gulfidan G. *et al.*	2022	Systems biomarkers for papillary thyroid cancer prognosis and treatment through multi-omics networks	10.1016/j.abb.2021.109085	Meta-analysis
Nikas I.P., Ryu H.S.	2022	The application of high-throughput proteomics in cytopathology	10.4132/jptm.2022.08.30	Review
Walton N.A. *et al.*	2022	The Development of an Infrastructure to Facilitate the Use of Whole Genome Sequencing for Population Health	10.3390/jpm12111867	No key words
Grimm D. *et al.*	2022	The Fight against Cancer by Microgravity: The Multicellular Spheroid as a Metastasis Model	10.3390/ijms23063073	Review
Pengzhou T. *et al.*	2022	The value of diagnostic nomogram based on CT radiomics for the preoperative differentiation between benign and malignant thyroid follicular neoplasms	10.3760/cma.j.cn112149-20210423-00403	No english

Di Cristofano A.	2022	The Year in Basic Thyroid Cancer Research	10.1089/thy.2021.0561	No key words
Imam S. *et al.*	2022	Thyroid Cancer Screening Using Tumor-Associated DN T Cells as Immunogenomic Markers	10.3389/fonc.2022.891002	No key words
Tarabichi M. *et al.*	2022	Thyroid cancer under the scope of emerging technologies	10.1016/j.mce.2021.111491	Review
Kim J.K. *et al.*	2022	Transcriptome-metabolome-wide association study (TMWAS) in rats revealed a potential carcinogenic effect of DEHP in thyroid associated with eicosanoids	10.1016/j.envres.2022.113805	No key words
Loberg M. *et al.*	2022	Transcriptomic Analysis Identifies a Subset of BRAF-Like Thyroid Cancers at Risk for Future Poor Outcome	10.1159/000527858	Conference Abstract
Loberg M. *et al.*	2022	Transcriptomic Analysis Identifies a Subset of BRAF-Like Thyroid Cancers at Risk for Future Poor Outcome	10.1016/j.jasc.2022.07.119	Conference Abstract
Li Y. *et al.*	2022	Transcriptomic signatures associated with autoimmune thyroiditis in papillary thyroid carcinoma and cancer immunotherapy-induced thyroid dysfunction	10.1016/j.csbj.2022.05.019	No key words
Tong Y. *et al.*	2022	Ultrasound-based radiomics analysis for preoperative prediction of central and lateral cervical lymph node metastasis in papillary thyroid carcinoma: a multi-institutional study	10.1186/s12880-022-00809-2	No key words
Jin P. *et al.*.	2022	Ultrasound-based radiomics nomogram combined with clinical features for the prediction of central lymph node metastasis in papillary thyroid carcinoma patients with Hashimoto's thyroiditis	10.3389/fendo.2022.993564	No key words
Shi Y. *et al.* F.	2022	Ultrasound-based radiomics XGBoost model to assess the risk of central cervical lymph node metastasis in patients with papillary thyroid carcinoma: Individual application of SHAP	10.3389/fonc.2022.897596	No key words
Hou X. *et al.*	2022	Unveiling the molecular features, relevant immune and clinical characteristics of SIGLEC15 in thyroid cancer	10.3389/fimmu.2022.975787	No key words

Alvarez A.G. *et al.*	2023	2211MO Uncovering the mechanisms of persistent disease in RET-altered thyroid cancers: Insights from patient-derived xenograft models treated with selective RET inhibitors	10.1016/j.annonc.2023.09.993	Conference Abstract
Huang J. *et al.*	2023	A study on the detection of thyroid cancer in Hashimoto's thyroiditis using computed tomography imaging radiomics	10.1016/j.jrras.2023.100677	No key words
Nourbakhsh M. *et al.*	2023	A workflow to study mechanistic indicators for driver gene prediction with Moonlight	10.1093/bib/bbad274	No key words
Yang S. *et al.*.	2023	Advances in transcriptomics and proteomics in differentiated thyroid cancer: An updated perspective (Review)	10.3892/ol.2023.13982	Review
Ruiz E. *et al.*	2023	An Integrative Multi-Omics Analysis of The Molecular Links between Aging and Aggressiveness in Thyroid Cancers	10.14336/AD.2022.1021	No key words
Xu H. *et al.*	2023	Analysis of preoperative computed tomography radiomics and clinical factors for predicting postsurgical recurrence of papillary thyroid carcinoma	10.1186/s40644-023-00629-9	No key words
Lu L. *et al.*	2023	Anaplastic transformation in thyroid cancer revealed by single-cell transcriptomics	10.1172/JCI169653	Review
Dondi F. *et al.*	2023	Application of radiomics and machine learning to thyroid diseases in nuclear medicine: a systematic review	10.1007/s11154-023-09822-4	Systematic Review
Wang C. *et al.*	2023	Artificial intelligence-based prediction of cervical lymph node metastasis in papillary thyroid cancer with CT	10.1007/s00330-023-09700-2	No key words
Kim N. *et al.*	2023	CCR8 as a Therapeutic Novel Target: Omics-Integrated Comprehensive Analysis for Systematically Prioritizing Indications	10.3390/biomedicines11112910	No key words
Toraih E.A. *et al.*	2023	Chromatin-Accessible miRNA Regulons Driving Thyroid Tumorigenesis and Progression	10.1097/XCS.0000000000000541	No free article
Dai L. *et al.*	2023	circAGTPBP1 promotes the progression of papillary thyroid cancer through the notch pathway via the miR-34a-5p/notch1 axis	10.1016/j.isci.2023.107564	No key words

Te Beek E.T. *et al.*	2023	Clinical Pharmacology of Radiotheranostics in Oncology	10.1002/cpt.2598	Review
Jiang L. *et al.*	2023	Clinical-Radiomics Nomogram Based on Contrast-Enhanced Ultrasound for Preoperative Prediction of Cervical Lymph Node Metastasis in Papillary Thyroid Carcinoma	10.3390/cancers15051613	No key words
Lee J. *et al.*	2023	Comparative Analysis of Driver Mutations and Transcriptomes in Papillary Thyroid Cancer by Region of Residence in South Korea	10.3803/EnM.2023.1758	No key words
Moon J. *et al.*	2023	Contrast-enhanced CT-based Radiomics for the Differentiation of Anaplastic or Poorly Differentiated Thyroid Carcinoma from Differentiated Thyroid Carcinoma: A Pilot Study	10.1038/s41598-023-31212-8	No key words
Haase J. *et al.*	2023	CREBBP/EP300 disruption promotes tumor progression and confers synthetic lethality in anaplastic thyroid cancers	10.1158/1538-7445.AM2023-3068	Conference Abstract
Wei T. *et al.*	2023	Development of a Clinical-Radiomics Nomogram That Used Contrast-Enhanced Ultrasound Images to Anticipate the Occurrence of Preoperative Cervical Lymph Node Metastasis in Papillary Thyroid Carcinoma Patients	10.2147/IJGM.S424880	No key words
Ren Y. *et al.*	2023	Dual-modal radiomics for predicting cervical lymph node metastasis in papillary thyroid carcinoma	10.3233/XST-230091	No key words
Volpi E.M. *et al.*	2023	Editorial: Recent advances in papillary thyroid carcinoma: Progression, treatment and survival predictors	10.3389/fendo.2023.1163309	Editorial
Xiong Y. *et al.*	2023	Evaluation of accuracy of pathological diagnosis based on thyroid core needle biopsy	10.19723/j.issn.1671-167X.2023.02.006	No english
Liu Y. *et al.*	2023	Exploration and validation of key genes associated with early lymph node metastasis in thyroid carcinoma using weighted gene co-expression network analysis and machine learning	10.3389/fendo.2023.1247709	No key words
Świrta J.S. *et al.*	2023	Expression of micro-ribonucleic acids in thyroid nodules and serum to discriminate between follicular adenoma and cancer in patients with a fine needle aspiration biopsy classified as suspicious for follicular neoplasm: A preliminary study	10.17219/acem/160003	Conference Abstract

Acuña-Ruiz A. *et al.*	2023	Genomic and epigenomic profile of thyroid cancer	10.1016/j.beem.2022.101656	Review
Minna E. *et al.*	2023	Genomic and transcriptomic analyses of thyroid cancers identify DICER1 somatic mutations in adult follicular-patterned RAS-like tumors	10.3389/fendo.2023.1267499	No key words
Lee S.E. *et al.*	2023	Genomic and transcriptomic characterization of medullary thyroid cancer	10.1158/1538-7445.AM2023-6069	Conference Abstract
Alzahrani A. *et al.*	2023	GERMLINE GENETIC VARIANTS IN APPARENTLY SPORADIC DIFFERENTIATED THYROID CANCER	10.1089/thy.2023.29161.lb.abstracts	Conference Abstract
Nabell L. *et al.*	2023	HB-200 arenavirus-based immunotherapy plus pembrolizumab as a first-line treatment of patients with recurrent/metastatic HPV16 positive head and neck cancer	10.1016/j.annonc.2023.09.2065	Conference Abstract
Feng Z. *et al.*	2023	Identification an innovative classification and nomogram for predicting the prognosis of thyroid carcinoma patients and providing therapeutic schedules	10.1007/s00432-023-05252-6	No key words
Cadena-Ullauri S. *et al.*	2023	Identification of KIT and BRAF mutations in thyroid tissue using next-generation sequencing in an Ecuadorian patient: A case report	10.3389/fonc.2022.1101530	No key words
Li Q. *et al.*	2023	Identification of Serpin peptidase inhibitor clade A member 1 (SERPINA1) might be a poor prognosis biomarker promoting the progression of papillary thyroid cancer	10.1016/j.lfs.2023.121938	No key words
Zhang W. *et al.*	2023	Integrated gene profiling of fine-needle aspiration sample improves lymph node metastasis risk stratification for thyroid cancer	10.1002/cam4.5770	No key words
Cararo Lopes E. *et al.*	2023	Integrated metabolic and genetic analysis reveals distinct features of human differentiated thyroid cancer	10.1002/ctm2.1298	No key words
Cararo-Lopes E. *et al.*	2023	Integrated metabolic and genetic analysis reveals distinct features of primary differentiated thyroid cancer and its metastatic potential in humans	10.1101/2023.03.09.23287037	No key words
Sun Z, Feng D. *et al.*	2023	Integrated proteomic and metabolomic analysis of plasma reveals regulatory pathways and key elements in thyroid cancer	10.1039/d3mo00142c	No key words
Itai Y. *et al.*	2023	Integration of gene expression and DNA methylation data across different experiments	10.1093/nar/gkad566	No key words

Ouyang J. *et al.*	2023	Integration of metabolomics and transcriptomics reveals metformin suppresses thyroid cancer progression via inhibiting glycolysis and restraining DNA replication	10.1016/j.biopha.2023.115659	No key words
Fan F. *et al.*	2023	Integration of ultrasound-based radiomics with clinical features for predicting cervical lymph node metastasis in postoperative patients with differentiated thyroid carcinoma	10.1007/s12020-023-03644-9	No key words
Sugier P.E. *et al.*	2023	Investigation of Shared Genetic Risk Factors Between Parkinson's Disease and Cancers	10.1002/mds.29337	No key words
Marcoux P. *et al.*	2023	iPSC: IMPACT OF THE OVEREXPRESSION OF THE TYROSINE KINASE (TK) RECEPTOR RET IN THE HEMATOPOIETIC POTENTIAL OF INDUCED PLURIPOTENT STEM CELLS (IPSC)	10.1016/S1465-3249(23)00694-1	Conference Abstract
Zhang W. *et al.*	2023	KNSTRN, a Poor Prognostic Biomarker, Affects the Tumor Immune Microenvironment and Immunotherapy Outcomes in Pan-Cancer	10.1155/2023/6729717	No key words
Emmett L. *et al.*	2023	LBA84 Enzalutamide and 177Lu-PSMA-617 in poor-risk, metastatic, castration-resistant prostate cancer (mCRPC): A randomised, phase II trial: ENZA-p (ANZUP 1901)	10.1016/j.annonc.2023.10.086	Conference Abstract
Zeng Z. *et al.*	2023	LncRNA HAGLROS contribute to papillary thyroid cancer progression by modulating miR-206/HMGA2 expression	10.18632/aging.205321	No key words
Leandro-García L.J., Landa I.	2023	Mechanistic Insights of Thyroid Cancer Progression	10.1210/endocr/bqad118	Review
Zhu H. *et al.*	2023	Models of ultrasonic radiomics and clinical characters for lymph node metastasis assessment in thyroid cancer: a retrospective study	10.7717/peerj.14546	No key words
Newbold K.	2023	Molecular genotyping in medullary thyroid cancer	10.1097/CCO.0000000000000915	Review
Xu G.J. *et al.*	2023	Molecular signature incorporating the immune microenvironment enhances thyroid cancer outcome prediction	10.1016/j.xgen.2023.100409	No key words
Sipos J.A., Ringel M.D.	2023	Molecular testing in thyroid cancer diagnosis and management	10.1016/j.beem.2022.101680	Review
Luvhengo T.E. *et al.*	2023	Multi-Omics and Management of Follicular Carcinoma of the Thyroid	10.3390/biomedicines11041217	Review
Li J. *et al.*	2023	Multiclassifier Radiomics Analysis of Ultrasound for Prediction of Extrathyroidal Extension in Papillary Thyroid Carcinoma in Children	10.7150/ijms.79758	No key words

Stenman A., Juhlin C.C.	2023	Novel Insights in the Genomics of Anaplastic Thyroid Carcinoma: A Role for Cyclin-Dependent Kinase Inhibition?	10.3390/cancers15184621	Editorial
Dhuli K. *et al.*	2023	Omics sciences and precision medicine in thyroid cancer	10.7417/CT.2023.2467	Review
Wang Y. *et al.*	2023	Pan-cancer analysis from multi-omics data reveals AAMP as an unfavourable prognostic marker	10.1186/s40001-023-01234-z	No key words
Hao J. *et al.*	2023	Pan-Cancer Study of the Prognosistic Value of Selenium Phosphate Synthase 1	10.1177/10732748231170485	No key words
Yan X. *et al.*	2023	Predicting central lymph node metastasis in patients with papillary thyroid carcinoma based on ultrasound radiomic and morphological features analysis	10.1186/s12880-023-01085-4	No key words
Jiang L. *et al.*	2023	Predicting Extrathyroidal Extension in Papillary Thyroid Carcinoma Using a Clinical-Radiomics Nomogram Based on B-Mode and Contrast-Enhanced Ultrasound	10.3390/diagnostics13101734	No key words
Peng Y. *et al.*	2023	Prediction of Central Lymph Node Metastasis in cN0 Papillary Thyroid Carcinoma by CT Radiomics	10.1016/j.acra.2022.09.002	No key words
Kong D. *et al.*	2023	Preliminary study on CT contrast-enhanced radiomics for predicting central cervical lymph node status in patients with thyroid nodules	10.3389/fonc.2023.1060674	No key words
Hu W. *et al.*	2023	Preoperative Cervical Lymph Node Metastasis Prediction in Papillary Thyroid Carcinoma: A Noninvasive Clinical Multimodal Radiomics (CMR) Nomogram Analysis	10.1155/2023/3270137	No key words
Wan F. *et al.*	2023	Preoperative prediction of extrathyroidal extension: radiomics signature based on multimodal ultrasound to papillary thyroid carcinoma	10.1186/s12880-023-01049-8	No key words
Fagin J.A., Nikiforov Y.E.	2023	Progress in Thyroid Cancer Genomics: A 40-Year Journey	10.1089/thy.2023.0045	No key words
Chung H.J. *et al.*	2023	Radiomics Analysis of Gray-Scale Ultrasonographic Images of Papillary Thyroid Carcinoma > 1 cm: Potential Biomarker for the Prediction of Lymph Node Metastasis	10.3348/jksr.2021.0155	No key words
Gao X.	2023	Radiomics approaches for predicting non-iodine-avid status of lung metastases in patients with differentiated thyroid cancer based on CT: a prospective observational study	10.1007/s00259-023-06333-x	Conference Abstract

HajiEsmailPoor Z. *et al.*	2023	Radiomics diagnostic performance in predicting lymph node metastasis of papillary thyroid carcinoma: A systematic review and meta-analysis	10.1016/j.ejrad.2023.111129	Systematic Review
Lu S. *et al.*	2023	Radiomics features from whole thyroid gland tissue for prediction of cervical lymph node metastasis in the patients with papillary thyroid carcinoma	10.1007/s00432-023-05184-1	No key words
Shen C. *et al.*	2023	Research on the oncogenic role of the house-keeping gene GAPDH in human tumors	10.21037/tcr-22-1972	No key words
Wu Y. *et al.*	2023	Systematic pan-cancer analysis of the potential tumor diagnosis and prognosis biomarker P4HA3	10.3389/fgene.2023.1045061	No key words
Wang Z. *et al.*	2023	Systemic pharmacology and bioinformatics: Exploring the modern biological mechanisms of rhubarb in the treatment of papillary thyroid carcinoma	10.1002/mef2.69	No key words
Jingtai Z. *et al.*	2023	Targeting Aurora-A inhibits tumor progression and sensitizes thyroid carcinoma to Sorafenib by decreasing PFKFB3-mediated glycolysis	10.1038/s41419-023-05709-z	No key words
Gu L. *et al.*	2023	The adaptive evolution of cancer driver genes	10.1186/s12864-023-09301-9	No key words
Li H. *et al.*	2023	THE IDENTIFICATION OF NOVEL GENETIC VULNERABILITIES IN ANAPLASTIC THYROID CANCER CELLS TO OVERCOME RESISTANCE TO BRAFV600E INHIBITOR USING A GENOME-WIDE CRISPR SCREEN	10.1089/thy.2023.29156.abstracts	Conference Abstract
Lu W.J. *et al.*	2023	Three-dimensional ultrasound-based radiomics nomogram for the prediction of extrathyroidal extension features in papillary thyroid cancer	10.3389/fonc.2023.1046951	No key words
Maurea S. *et al.*	2023	Thyroid Cancer Radiomics: Navigating Challenges in a Developing Landscape	10.3390/cancers15245884	Editorial
Dai Q. *et al.*	2023	Ultrasound radiomics models based on multimodal imaging feature fusion of papillary thyroid carcinoma for predicting central lymph node metastasis	10.3389/fonc.2023.1261080	No key words
Zhang M. *et al.*	2023	Ultrasound radiomics nomogram for predicting large-number cervical lymph node metastasis in papillary thyroid carcinoma	10.3389/fonc.2023.1159114	No key words
Hung S.-H. *et al.*	2023	Utilizing T cell receptor-based therapy to treat anaplastic thyroid cancer	10.1158/1557-3265.AACRAHNS23-PO-061	Conference Abstract

Xue J. *et al.*	2023	Value of clinical features combined with multimodal ultrasound in predicting lymph node metastasis in cervical central area of papillary thyroid carcinoma	10.1002/jcu.23465	No key words
Döring C. *et al.*	2023	Whole-exome sequencing of calcitonin-producing pancreatic neuroendocrine neoplasms indicates a unique molecular signature	10.3389/fonc.2023.1160921	No key words
Lee S.E. *et al.*	2024	Unraveling the role of the mitochondrial one-carbon pathway in undifferentiated thyroid cancer by multi-omics analyses	10.1038/s41467-024-45366-0	No key words
Doostmohammadi A. *et al.*	2024	Potentials and future perspectives of multi-target drugs in cancer treatment: the next generation anti-cancer agents	10.1186/s12964-024-01607-9	Review
Li Z. *et al.*	2024	A CT based radiomics analysis to predict the CN0 status of thyroid papillary carcinoma: a two- center study	10.1186/s40644-024-00690-y	No key words
Jin X. *et al.*	2024	Revolutionary multi-omics analysis revealing prognostic signature of thyroid cancer and subsequent in vitro validation of SNAI1 in mediating thyroid cancer progression through EMT	10.1007/s10238-024-01387-z	No key words
Jung S. *et al.*	2024	CancerGATE: Prediction of cancer-driver genes using graph attention autoencoders	10.1016/j.compbiomed.2024.108568	No key words
Wang Z. *et al.*	2024	Interactions between LAMP3+ dendritic cells and T-cell subpopulations promote immune evasion in papillary thyroid carcinoma	10.1136/jitc-2024-008983	No key words
Cao H. *et al.*	2024	Prognostic analysis of 131I efficacy after papillary thyroid carcinoma surgery based on CT radiomics	10.1210/clinem/dgae364	No key words
Sun H. *et al.*	2024	Multi-omics analysis-based macrophage differentiation-associated papillary thyroid cancer patient classifier	10.1016/j.tranon.2024.101889	No key words
Yang Y. *et al.*	2024	Massively Parallel CAR-T Cell Phenotyping Enables Identification of High Efficiency Candidates	10.1016/j.ymthe.2024.04.020	Conference Abstract
Liu Z. *et al.*	2024	Combining radiomics with thyroid imaging reporting and data system to predict lateral cervical lymph node metastases in medullary thyroid cancer	10.1186/s12880-024-01222-7	No key words
Nourbakhsh M. *et al.*	2024	Revealing cancer driver genes through integrative transcriptomic and epigenomic analyses with Moonlight	10.1101/2024.03.14.584946	No key words
Lu Y. *et al.*	2024	Prognostic significance and immunological role of HPRT1 in human cancers	10.17305/bb.2023.9775	No key words
Sakthikumar S. *et al.*	2024	Genomic analysis across 53 canine cancer types reveals novel mutations and high clinical actionability potential	10.1111/vco.12944	No key words

Yan K. *et al.*	2024	Spatial transcriptomics reveals prognosis-associated cellular heterogeneity in the papillary thyroid carcinoma microenvironment	10.1002/ctm2.1594	No key words
Hung S.-H. *et al.*	2024	Utilizing T cell receptor-based therapy to treat anaplastic thyroid cancer	10.1158/1538-7445.AM2024-24	Conference Abstract
Zeng P. *et al.*	2024	Understanding and overcoming innate and acquired resistance to type I and II RAF inhibitors in anaplastic thyroid cancer using translational functional genomics	10.1158/1538-7445.AM2024-3580	Conference Abstract
Chantadisai M. *et al.*	2024	Combined clinical variable and radiomics of post-treatment total body scan for prediction of successful I-131 ablation in low-risk papillary thyroid carcinoma patients	10.1038/s41598-024-55755-6	No key words
Wang Z. *et al.*	2024	Multi-omics clustering analysis carries out the molecular specific subtypes of thyroid carcinoma: implicating for the precise treatment strategies	10.1101/2024.02.25.24303184	No key words
Tourneur A. *et al.*	2024	Single nuclei and spatial transcriptomes suggest a stratification of papillary and anaplastic thyroid cancer cells	10.1101/2024.02.15.580495	No key words
Peng Y. *et al.*	2024	The Application of Artificial Intelligence in Thyroid Nodules: A Systematic Review Based on Bibliometric Analysis	10.2174/0118715303264254231117113456	Systematic Review
Modica R. *et al.*	2024	Current understanding of pathogenetic mechanisms in neuroendocrine neoplasms	10.1080/17446651.2023.2279540	Review
Shen B. *et al.*	2024	Ultrasound-based Radiomics for Predicting Metastasis in the Lymph Nodes Posterior to the Right Recurrent Laryngeal Nerve in Patients with Papillary Thyroid Cancer	10.2174/0115734056257332231024112410	No key words
Peliciari-Garcia R.A. *et al.*	2024	Multi-omics Investigations in Endocrine Systems and Their Clinical Implications	10.1007/978-3-031-50624-6_10	Book Chapter
Li M.H. *et al.*	2024	Prediction of cervical lymph node metastasis in solitary papillary thyroid carcinoma based on ultrasound radiomics analysis	10.3389/fonc.2024.1291767	No key words
Cui Z. *et al.*	2024	From genomic spectrum of NTRK genes to adverse effects of its inhibitors, a comprehensive genome-based and real-world pharmacovigilance analysis	10.3389/fphar.2024.1329409	No key words
Yamazaki H. *et al.*	2024	Genetic landscape of 482 thyroid carcinomas: analysis with the national datacenter for cancer genomic medicine in Japan	10.1007/s12020-024-03738-y	No key words
Liu Q. *et al.*	2024	Multi-modal ultrasound multistage classification of PTC cervical lymph node metastasis via DualSwinThyroid	10.3389/fonc.2024.1349388	No key words

Robert Frost H.	2024	Tissue-adjusted pathway analysis of cancer (TPAC): A novel approach for quantifying tumor-specific gene set dysregulation relative to normal tissue	10.1371/journal.pcbi.1011717	No key words
Feng J.-W *et al.*	2024	Development and Validation of Clinical-Radiomics Nomogram for Preoperative Prediction of Central Lymph Node Metastasis in Papillary Thyroid Carcinoma	10.1016/j.acra.2023.12.008	No key words
Saito Y. *et al.*	2024	Comprehensive genomic profiling from C-CAT database unveiled over 80% presence of oncogenic drivers in anaplastic thyroid carcinoma including BRAF, RAS family, NF1, and FGFR1	10.1111/cen.15098	No key words
Huang Y *et al.*	2024	LASS2 suppresses metastasis in multiple cancers by regulating the ferroptosis signalling pathway through interaction with TFRC	10.1186/s12935-024-03275-8	No key words
Qu N. *et al.*	2024	Integrated proteogenomic and metabolomic characterization of papillary thyroid cancer with different recurrence risks	10.1038/s41467-024-47581-1	No key words
Han X. *et al.*	2024	Association of cancer and schizophrenia, major depression and bipolar disorder: A Mendelian randomization study	10.1016/j.jpsychores.2024.111806	No key words
Lv X. *et al.*	2024	Prediction of lymph node metastasis in patients with papillary thyroid cancer based on radiomics analysis and intraoperative frozen section analysis: A retrospective study	10.1111/coa.14162	No key words
Candia J., Ferrucci L.	2024	Assessment of Gene Set Enrichment Analysis using curated RNA-seq-based benchmarks	10.1371/journal.pone.0302696	No key words
Chopyk D.M. *et al.*	2024	Strategies to investigate migration and metastases in thyroid cancer	10.1016/j.coemr.2023.100502	Review
Zuazo C.E. *et al.*	2024	Bioinformatic analysis of an annotated genomic database is clinically useful in a private cancer center	10.1158/1538-7445.AM2024-4972	Conference Abstract
Mu J. *et al.*	2024	Prediction of cervical lymph node metastasis in differentiated thyroid cancer based on radiomics models	10.1093/bjr/tqae010	Meta-analysis
Abooshahab R. *et al.*	2024	Thyroid cancer cell metabolism: A glance into cell culture system-based metabolomics approaches	10.1016/j.yexcr.2024.113936	Review
Kim Y.H. *et al.*	2024	Integrative Multi-omics Analysis Reveals Different Metabolic Phenotypes Based on Molecular Characteristics in Thyroid Cancer	10.1158/1078-0432.CCR-23-2025	Review
Dondi F. *et al.*	2024	Application of radiomics and machine learning to thyroid diseases in nuclear medicine: a systematic review	10.1007/s11154-023-09822-4	Review
Pitoia F., Trimboli P.	2024	New insights in thyroid diagnosis and treatment	10.1007/s11154-023-09859-5	Review

Candia J., Ferrucci L.	2024	Assessment of Gene Set Enrichment Analysis using curated RNA-seq-based benchmarks	10.1101/2024.01.10.575094	No key words
Tan J.K. *et al.*	2024	Exploring the advances of single-cell RNA sequencing in thyroid cancer: a narrative review	10.1007/s12032-023-02260-x	Review
Wang Y. *et al.*	2024	Exosome-Mediated Communication in Thyroid Cancer: Implications for Prognosis and Therapeutic Targets	10.1007/s10528-024-10833-2	No key words
Sun H. *et al.*	2024	Prioritization of drug targets for thyroid cancer: a multi-omics Mendelian randomization study	10.1007/s12020-024-03933-x	No key words
Zhang S. *et al.*	2024	Ultrasound-Base Radiomics for Discerning Lymph Node Metastasis in Thyroid Cancer: A Systematic Review and Meta-analysis	10.1016/j.acra.2024.03.012	Systematic Review
Turner J.H.	2024	Theranostics: Timing is Everything	10.1089/cbr.2024.0088	Editorial
DeSouza N.R. *et al.*	2024	Long Non-Coding RNAs as Determinants of Thyroid Cancer Phenotypes: Investigating Differential Gene Expression Patterns and Novel Biomarker Discovery	10.3390/biology13050304	Review
Ju S.H. *et al.*	2024	Transcriptomic characteristics according to tumor size and SUV(max) in papillary thyroid cancer patients	10.1038/s41598-024-61839-0	No key words
Lee S.E. *et al.*	2024	Neutrophil diversity is associated with T-cell immunity and clinical relevance in patients with thyroid cancer	10.1038/s41420-024-01970-z	No key words
Gulec S.A., Meneses E.	2024	Theranostic Risk Stratification for Thyroid Cancer in the Genomic Paradigm	10.3390/cancers16081585	Review
Guan Z. *et al.*	2024	A Cuproptosis-Related gene Signature as a Prognostic Biomarker in Thyroid Cancer Based on Transcriptomics	10.1007/s10528-024-10767-9	No key words
Zeng P.Y.F. *et al.*	2024	The genomic and evolutionary landscapes of anaplastic thyroid carcinoma	10.1016/j.celrep.2024.113826	No key words
Pires C. *et al.*	2024	Identification of Germline FOXE1 and Somatic MAPK Pathway Gene Alterations in Patients with Malignant Struma Ovarii, Cleft Palate and Thyroid Cancer	10.3390/ijms25041966	No key words
Fan X. *et al.*	2024	Diagnosing postoperative lymph node metastasis in thyroid cancer with multimodal radiomics and clinical features	10.1177/20552076241233244	No key words
Jin Z. *et al.*	2024	Prognosis and therapy in thyroid cancer by gene signatures related to natural killer cells	10.1002/jgm.3657	No key words

Jones S.	2024	Looking back over 2023 and welcome to the 21(st) issue of Personalized Medicine	10.2217/pme-2023-0147	No free article
Zhu Y. *et al.*	2024	Deep learning radiomics of multimodal ultrasound for classifying metastatic cervical lymphadenopathy into primary cancer sites: a feasibility study	10.1055/a-2161-9369	No free article
Tarasova V.D. *et al.*	2024	Characterization of the Thyroid Cancer Genomic Landscape by Plasma-Based Circulating Tumor DNA Next-Generation Sequencing	10.1089/thy.2023.0204	No key words
Chen M. *et al.*	2024	Dual-Energy Computed Tomography in Detecting and Predicting Lymph Node Metastasis in Malignant Tumor Patients: A Comprehensive Review	10.3390/diagnostics14040377	Review
Condello V. *et al.*	2024	Spatial Transcriptomics in a Case of Follicular Thyroid Carcinoma Reveals Clone-Specific Dysregulation of Genes Regulating Extracellular Matrix in the Invading Front	10.1007/s12022-024-09798-0	No key words
Ivanova D.	2024	BIG BIOMEDICAL DATA ANALYTICS IN SUPPORT OF PRECISION MEDICINE	10.55630/mem.2024.53.018-024	Review
Han N.A. *et al.*	2024	APPLICATION of ARTIFICIAL INTELLIGENCE in the DIAGNOSIS of THYROID CANCER with ENHANCED COMPUTED TOMOGRAPHY	10.1142/S0219519424400177	No key words

N/A, Not Available.
