# Supplementary material for: Association of radiomic features with genomic signatures in thyroid cancer: a systematic review
Source: J Transl Med. 2024 Nov 30;22:1088. doi: 10.1186/s12967-024-05896-z (PMC11608493; doi:10.1186/s12967-024-05896-z)
Supplement: Supplementary file 4 — Supplementary material 4. Description of radiomic features reported in the studies analyzed. [file 12967_2024_5896_MOESM4_ESM.pdf]

Additional file 4: Description of radiomic features reported in the studies analyzed.

**Authors (year)**	**Imaging modality**	**Radiomic features**	**Radiomic features analysis**
Dong *et al.* (2023)	CT	First-order; GLSZM; NGTDM; GLRLM; GLDM; GLCM	SelectKBest; mRMR; LASSO
Tong *et al.* (2021)	US	GLCM; GLRLM	LASSO
Zheng *et al.* (2023)	MRI	First-order; GLCM; GLRLM; GLSZM; GLDM; LoG transformed; Wavelet trasformed	mRMR
Wang *et al.* (2022)	US	Grayscale	mRMR; LASSO
Yoon *et al.* (2020)	US	First-order; GLCM; GLRLM	LASSO
Yu *et al.* (2022)	US	First-order	LASSO
Zhang *et al.* (2024)	US	Morphological features; First-order; Second-order; Wavelet	LASSO

Abbreviation: CT, computed tomography; **GLCM,** gray level co-occurrence matrix; **GLDM**, gray level dependence matrix; **GLRLM,** gray level run length matrix; **GLSZM**, gray level size zone matrix; **LASSO**, least absolute shrinkage and selection operator; **MRI**, magnetic resonance imaging; **mRMR**, minimum redundancy maximum relevance; **NGTDM**, neighborhood gray tone difference matrix; **US,** ultrasonography.
